# Empirical research on requirements quality: a systematic mapping study

**DOI:** 10.1007/s00766-021-00367-z

**Published:** 2022-02-15

**Authors:** Lloyd Montgomery, Davide Fucci, Abir Bouraffa, Lisa Scholz, Walid Maalej

**Affiliations:** 1 University of Hamburg, Hamburg, Germany; 2 Blekinge Tekniska Högskola, Karlskrona, Sweden

**Keywords:** Systematic mapping study, Secondary study, Requirements quality, Empirical research

## Abstract

Research has repeatedly shown that high-quality requirements are essential for the success of development projects. While the term “quality” is pervasive in the field of requirements engineering and while the body of research on requirements quality is large, there is no meta-study of the field that overviews and compares the concrete quality attributes addressed by the community. To fill this knowledge gap, we conducted a systematic mapping study of the scientific literature. We retrieved 6905 articles from six academic databases, which we filtered down to 105 relevant primary studies. The primary studies use empirical research to explicitly define, improve, or evaluate requirements quality. We found that empirical research on requirements quality focuses on improvement techniques, with very few primary studies addressing evidence-based definitions and evaluations of quality attributes. Among the 12 quality attributes identified, the most prominent in the field are ambiguity, completeness, consistency, and correctness. We identified 111 sub-types of quality attributes such as “template conformance” for consistency or “passive voice” for ambiguity. Ambiguity has the largest share of these sub-types. The artefacts being studied are mostly referred to in the broadest sense as “requirements”, while little research targets quality attributes in specific types of requirements such as use cases or user stories. Our findings highlight the need to conduct more empirically grounded research defining requirements quality, using more varied research methods, and addressing a more diverse set of requirements types.

## Introduction

Requirements engineering (RE) is “the process of defining, documenting, and maintaining requirements” [1], where “requirements” designate real-world goals for, functions of, and constraints on systems [2]. The *quality* of requirements refers to the individual characteristics of requirements that both lead to a successful and cost-effective system, and solves the user’s needs [3]. Requirements quality depends on multiple factors such as the requirements author, the stakeholders consulted [4], the templates and processes used [5], and the verification processes followed [6]. Requirements quality research focuses on specific attributes of requirements, including ambiguity, completeness, consistency, complexity, and verifiability [3, 7–9].

The 1994 Standish Chaos Report described the perceived importance of requirements on software project success and failure [10]. The report details project success factors such as a “clear statement of requirements” and “clear vision and objectives”, as well as project failure factors such as “incomplete requirements” and “changing requirements and specifications” [10]. In 2007, Kamata and Tamai empirically validated the claims of the Standish Chaos Report through an investigation of 32 industrial software projects focusing on the impact of RE on quality, time, and cost calculations [11]. Their research found that a relatively small set of requirements have a strong impact on project success and failure, and projects within time and cost had an acceptable level of quality across all sections of the requirements documents. More recently, a 2017 survey of 136 organisations found that inconsistent, under-specified, and incomplete requirements are consistently rated among the top five reasons for project failures [12].

Requirements quality research addresses these aforementioned aspects in an attempt to increase project successes and limit project failures. The amount of research on this topic leads to key meta-questions such as which requirements quality attributes have primarily been investigated, which empirical methods have been used, and what kind of contexts (such as projects, participants, and documents) have been studied? Despite the broad interest in the field and the existence of multiple industry standards [8, 9, 13], to the best of our knowledge, no secondary study addressing the full breadth of requirements quality has been published. Pekar et al. provided a brief 4-page systematic mapping study in 2014 of requirements quality [14]. Heck and Zaidman conducted a systematic literature review in 2018 on quality criteria for requirements specifications [15], focusing only on agile software development. Zhao et al. conducted an SMS in 2020 on the use of natural language processing (NLP) for RE [16], limiting their search to articles utilising NLP. Although these secondary studies have approached the area of requirements quality, there is a lack of a general overview. Researchers looking to work on requirements quality and practitioners interested in understanding the state requirements quality have no single starting place in the literature.

To provide a comprehensive overview of the research on requirements quality, we performed a systematic mapping study (SMS) covering several contexts, quality attributes, quality improvement techniques, and RE activities. In particular, we collect *empirical research* on requirements quality that aims to understand or improve the quality of requirements *artefacts*. We retrieved 6905 articles from six academic databases. Applying multiple inclusion and exclusion criteria reduced the articles down to 105 primary studies for our SMS. Two of the authors read each of these articles and independently answered 18 questions, including who are the authors, what quality attributes are they addressing, what methods are they using, what RE activity are they targeting, and what tools have been created to assess and improve the quality of requirements. Our findings and discussions serve as a starting place for future empirical requirements quality research. Our full data set (articles, extracted data, and mapping scripts) is available for download in our replication package.^1^

The remainder of the paper is structured as follows. Section 2 discusses existing meta-studies in the area of requirements quality. Section 3 introduces the research questions and methodological details of our SMS. Section 4 presents our findings, which we then discuss in Sect. 5. Finally, Sect. 6 outlines the study validity and Sect. 7 concludes the work.

## Background

Secondary studies in RE focus on specific domains (e.g. Agile [17]), activities (e.g. elicitation [18], maintenance [19], testing [20]), tasks (e.g. stakeholder selection [21], goal-oriented process mining [22–24]), types of requirements (e.g. functional [25]), technologies used for RE (e.g. decision support systems [26]), human aspects (e.g. personality [27], culture [28]), and even empirical methods [29]. Here, we discuss a few secondary studies that target requirements quality.

Pekar at al. conducted an SMS that provides a generic overview of 67 studies, published between 1998 and 2013 [14]. They showed that requirements ambiguity, completeness, and correctness are the most investigated problems in RE research. They also show that the literature focuses on linguistic-based techniques for solving ambiguity, consistency checking, alternative perspectives from stakeholders, as well as general frameworks for requirements specification quality assessment.

Heck and Zaidman conducted an SMS that covered eleven primary studies, published between 2001 and 2014, investigating state-of-the-art research into quality of specifications in the context of agile software development [15]. They showed that the main qualities of specifications to take into account are completeness, uniformity, consistency, and correctness. For correctness, the practitioners’ literature (e.g. [30, 31]) proposed a set of quality criteria, the INVEST model [32], which is limited to user stories.

Zhao et al. conducted an SMS in 2020 regarding the application of NLP for RE covering 404 primary studies published between 1983 and 2019, investigating several aspects such as technologies, practices, and activities, [16]. They found that most primary studies target the RE analysis activity, whereas validation and verification—for which quality plays an important role—are the least targeted. The authors show that specification documents are the most studied in the RE research dealing with NLP, while user feedback and other user-generated content are lately getting more attention. The main NLP tasks performed in the context of RE activities are detection, extraction, and classification of information within a requirement document, as well as traceability.

The NaPiRE survey conducted with 136 diverse organisations around the world collected evidence of the industrial relevance for *comprehensive* requirements quality [12]. The results show that inconsistent, under-specified, and incomplete requirements are regularly rated among the top 5 causes for project failures. Badly written requirements were also reported as one of the problems organisations are struggling with the most.

Our SMS gives a *comprehensive* overview of empirical research on requirements quality in RE by covering different quality attributes, research methods, and contexts.

## Study design

### Research questions

Our goal is to map the field of empirical requirements quality research to offer interested researchers and practitioners an overview of the state of research, and offer insights towards future work. With this goal in mind, we follow Kitchenham and Charters [33] in creating multiple broad research questions, Napoleão et al. [34] in creating research questions that consider only population and intervention questions, and Petersen et al. [35, 36] who provided many examples of research question formats for SMSs. These considerations have led to the following research questions:

1. Empirical requirements quality research: Who is publishing in this field, where, when, and on which quality attributes?
2. Which empirical research methods are used to study requirements quality?
3. Which artefacts, activities, and tools have been studied in empirical requirements quality research?

### Research method

**Fig. 1:**
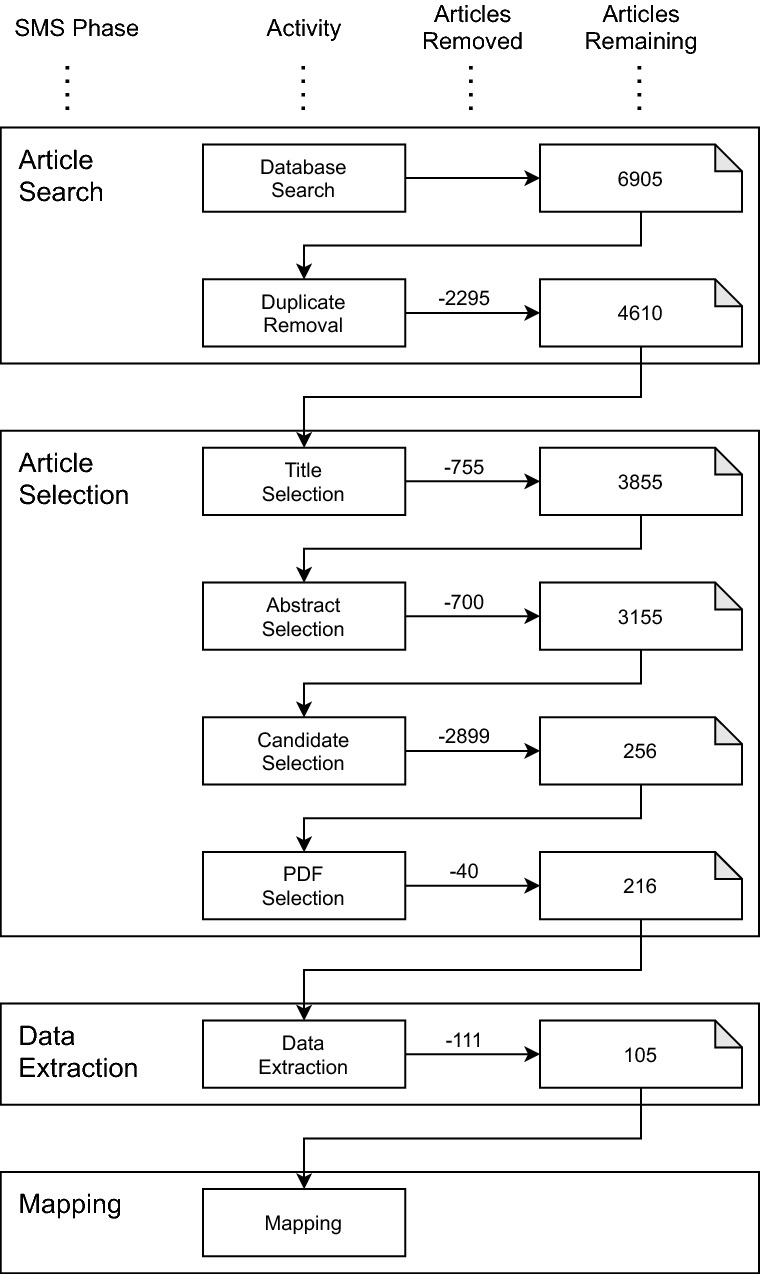
SMS review methods and number of primary studies at each phase and activity

To report on our research method, we follow the recommended structure of systematic reviews by Petersen et al [36] and Kitchenham and Charters [33]. This includes four phases: article search, article selection, data extraction, and mapping. We visualise the phases and activities in Fig. 1.

#### Article search

In the article search phase, we collected the primary studies for this SMS. This included two activities: database search and duplicate removal.

***Database search.*** The “database search” strategy is the most common search strategy in systematic reviews in software engineering (SE) [35]. We selected the following databases as primary sources: ACM Digital Library^2^, IEEE Xplore^3^, Elsevier ScienceDirect^4^, and SpringerLink^5^ Additionally, we selected two indexing systems: Web of Science^6^ and Google Scholar^7^ We selected these sources using the recommendations of Dyba et al. [37], Kitchenham and Brereton [38], and Petersen et al. [35] in using ACM, IEEE, and two indexing systems. We expanded on their recommendation to include Elsevier and Springer as these four (including ACM and IEEE) “host the major journals and conference proceedings related to SE and RE” [16].

**Table 1:** Search strings for each data source and number of results downloaded

Search source	Search string	Results
ACM Digital	(“requirements engineering” AND (“requirements quality” OR “quality of requirements” OR “issue quality”	106
Library	OR “quality of issues” OR “specification quality” OR “quality of specification”))	
IEEE Xplore	((“quality of issues” OR “requirements quality” OR “quality of requirements” OR “issue quality” OR “quality of specification” OR “specification quality”) AND “requirements engineering”)	682
ScienceDirect	(“requirements engineering” AND (“requirements quality” OR “quality of requirements” OR “issue quality” OR “quality of issues” OR “specification quality” OR “quality of specification”))	251
SpringerLink	(“requirements engineering” AND (“requirements quality” OR “quality of requirements” OR “issue quality” OR “quality of issues” OR “specification quality” OR “quality of specification”))	470
Web of Science	TS=(“requirements engineering” AND (“requirements quality” OR “quality of requirements” OR “issue quality” OR “quality of issues” OR “specification quality” OR “quality of specification”))	56
Google Scholar	“requirements engineering” “quality of issues” OR “requirements quality” OR “quality of requirements” OR “issue quality” OR “quality of specification” OR “specification quality”	5340
Total		6905

We targeted every study that addresses “requirements” and “quality”. However, in a broad sense, all RE research has the aim of improving the quality of *something*, whether through understanding or intervention. For this reason, we require the primary studies to explicitly address the quality of requirements by mentioning “requirements quality” or “quality of requirements”. All aspects of requirements quality and artefact types are of interest to this SMS; therefore, no restrictions were made in the search string.

To keep the results in scope, our search string first includes the term “requirements engineering”. Without this term, preliminary investigations revealed 3-10x more search results, most of which appeared to be from other research fields such as business and economics^8^. Second, we chose six variations of the term “requirements quality” to account for the power set of 1) synonymous for requirements (i.e. requirements, issues, and specification) and 2) two ways to refer to quality (X quality and quality of X). This resulted in the following search terms: “requirements quality”, “quality of requirements”, “issue quality”, “quality of issues”, “specification quality”, and “quality of specification”. The final search string was “(A and (B or C or D or E or F or G))” where A is “requirements engineering” and B–G are the six terms defined in the previous sentence.

Table 1 shows the search strings we used to download the data from each source (depending on the allowed syntax), as well as the results counts. The search string was applied to the metadata in all six searches, and the full text in all but Web of Science. In total, we obtained 6905 articles from the data sources, as shown in Fig. 1.

***Duplicate removal.*** To handle duplicates, we used the Mendeley^9^ “Check for Duplicates” feature which groups the articles into possible duplicate groups. The ultimate decision regarding the duplicate entries was manually resolved by the first author. In most cases, the title, authors, year, and abstract were all identical, leading to an obvious acceptance of the duplication. In cases of conflict between one of the aforementioned details, we retrieved the article and manually checked the details. We performed the duplicate removal activity on the 6905 articles from the database search activity, removing 2295 articles and leaving 4610 articles remaining.

#### Article selection

In the article selection phase, the first and second author (R1 and R2^10^) applied the inclusion/exclusion criteria to refine the list of primary studies. The article selection phase has four activities: title selection, abstract selection, candidate selection, and PDF selection. These four activities reduced the number of articles to 3855, 3155, 256, and 216, respectively. Following our research objectives, our inclusion and exclusion criteria were as follows.

**Inclusion criteria**

1. Peer-reviewed
2. Empirical Research

-
  - Surveys, experimental (including protocol studies), case studies, etc.
- **I3** Explicitly mentions the empirical definition, improvement, or evaluation of some quality attributes of requirements engineering artefacts, which are listed separately as follows:
  - Mentions any *definition* of requirements artefact quality
  - Mentions any *improvement* of requirements artefact quality
  - Mentions any *evaluation* of previous requirements artefact quality work

**Exclusion criteria**

- **E1** Non-English texts
- **E2** Article types
  - Position articles
  - Experience reports
  - Base articles/studies (an article that has been superseded by another article in this SMS)
  - Secondary/tertiary studies (literature surveys, SMS, etc.)
  - Books, book chapters, standards, and non-peer-reviewed articles
- **E3** Discuss RE together with other software development life cycle activities, while RE is not the focus

***Title selection.*** R1 applied the exclusion criteria to the article titles. We did this to filter search results which should not have been returned in the database search, such as non-English titles. This activity was only performed by R1 because it was a preliminary exclusion process, and the only articles rejected were obvious rejections where the title of the article described itself as a “secondary study”, “thesis”, or “vision paper”. We performed this activity on the 4610 articles from the article search phase, removing 755 articles and leaving 3855 articles remaining.

*Abstract selection* R1 applied the exclusion criteria to the article abstracts, removing obvious rejections based on information seen in the abstracts. This includes non-English articles, position papers, secondary studies, books, book chapters, and theses. Similar to the title selection activity above, this process was only performed by R1 due to the simplicity of the task (articles describing themselves as a “thesis” in the abstract are clear exclusions that do not require consensus). We performed this activity on the 3855 articles from the title selection activity, removing 700 articles and leaving 3155 articles remaining.

***Candidate selection.*** R1 and R2 read the title and abstract of each article and then independently labelled each as “include”, “exclude”, or “unsure”, following the guidelines of Petersen et al. [35]. R1 and R2 then reviewed the labels, settling disagreements through open discussions.

We evaluated the reliability of our labelling process using interrater reliability (IRR). We report per cent agreement [39, 40], Cohen’s Kappa [41], and S-Score [42]. Per cent agreement is reported for its simplicity, but the literature consistently warns against using it because it does not take into account the change agreement of the labelling task [41, 43, 44]. Cohen’s Kappa (1960) [41] is reported for its common usage as an IRR metric; however, our data set suffers from the prevalence problem due to the nature of secondary studies having broad search strings, which can cause Cohen’s Kappa to substantially misrepresent the IRR of a measure [43]. While some say “Cohen’s Kappa should *not* be calculated in such a situation” [45], we show it for completeness as other researchers argue that bias and prevalence should be included when discussing IRR [46]. Finally, we report Bennett et al.’s S-Score [42] which presents a much more realistic IRR measure when dealing with prevalence at the extremes. To interpret our S-Scores, we refer to Regier et al. [47], as they have a granular and up-to-date interpretation. Table 2 details these three IRR measures for each stage of the candidate selection.

R1 and R2 performed two trial runs prior to the full candidate selection activity to align their understanding of the candidate selection activity. In the first trial run, they labelled 50 articles at random from the 3155 remaining articles, followed by an alignment discussion to resolve disagreements. The first trial run had an S-Score [42] of 67%, which is considered “Very Good” [47]. In the second trial run, the raters again labelled 50 random articles, followed by an alignment discussion. The second trial run had an S-Score [42] of 73%, which is considered “Very Good” [47]. Both raters agreed that the process was refined enough to proceed with a full run.

R1 and R2 conducted the full candidate selection run. They labelled 3155 articles, with an S-Score [42] of 72.47%, which is “Very Good” [47]. They made a clear “include” or “exclude” decision regarding each article prior to the data extraction phase. The raters discussed all disagreements and came to a consensus for each article. There were three agreement strategies to assign the final labels. (1) For articles on which both researchers agreed and labelled “include” or “exclude”, the agreed-upon label was accepted. (2) For articles on which both researchers agreed and labelled “unsure”, they jointly agreed on “include” or “exclude” following an investigation of the article itself. (3) For articles with labelling disagreements, a process similar to (2) was conducted, taking into account the incoming bias held by each researcher. Regardless of the bias, they had an open discussion and agreed on final labels. In situations where they could not reach a confident and comfortable agreement, they chose the “include” label to allow for a more detailed investigation in the following phases. We applied the candidate selection activity on the 3155 articles from the abstract selection activity, removing 2899 articles and leaving 256 articles remaining (see Fig. 1).

***PDF selection.*** R1 removed obvious rejections based on information found while gathering the article PDFs, for example, downloading a 400-page PhD thesis. We performed this activity on the 256 articles from the candidate selection activity, removing 40 articles and leaving 216 articles remaining (see Fig. 1).

**Table 2:** Interrater agreement for candidate selection, trial runs and full run

Interrator agreement							
Trial Run 1							
		R2			Papers Labelled		50
		I	U	E	Post-alignment % Included		38.00%
R1	I	14	0	5	IRR	Percent agreement *	78.00%
	U	0	0	0		Cohen’s Kappa $\dag$	53.78%
	E	6	0	25		S Score $\ddag$	67.00%
Trial Run 2							
		R2			Papers Labelled		50
		I	U	E	Post-alignment % Included		20.00%
R1	I	6	1	3	IRR	Percent agreement *	82.00%
	U	0	0	0		Cohen’s Kappa $\dag$	49.44%
	E	3	2	35		S Score $\ddag$	73.00%
Full Run							
		R2			Papers Labelled		3155
		I	U	E	Post-alignment % Included		8.11%
R1	I	93	20	277	IRR	Percent agreement *	81.65%
	U	11	3	28		Cohen’s Kappa $\dag$	18.97%
	E	185	58	2480		S Score $\ddag$	72.47%

Include (I), Unsure (U), Exclude (E)

*[39, 40], $$\dag$$ [41], $$\ddag$$ [42]

#### Data extraction

The data extraction phase was conducted by the first four authors. R1 extracted data from all of the articles, while each of the other three authors extracted from a subset of the articles (88, 60, and 68, respectively). This resulted in each of the papers being peer-labelled by two authors. Through this in depth process of extracting data, more exclusions were discovered. Many of these exclusions were due to a lack of empiricism when reviewing the full article. We performed the data extraction activity on the 216 articles from the Article Selection phase, removing 111 articles and leaving 105 articles remaining (see Fig. 1). This was the final reduction activity, thus arriving at our final number of 105 primary studies.

The process of reading the articles and extracting the data was done independently by each researcher. Once extracted, the researchers met to discuss the extracted questions, and resolve disagreements. The dispute resolution process involved opening up the original article, discussing the disputed question(s) in context, and coming to an agreement. To mitigate potential issues with fatigue, each meeting to discuss extracted data and resolve disputes was no longer than 60 minutes, with a short break after 30 minutes. There are three data extraction categories that map directly on to the three research questions. Each data extraction category has a number of extraction questions, as listed in Table 3. For each extraction question, a coding style and extraction style were pre-determined based on the desired outcome of the extraction.

**Table 3:** Data extraction categories and questions

Extraction	Extraction question	Code	Extract
Category		Style	Style
Who, What, Where, and When	Authors	O	S
	Publishing year	C	S
	Venue	O	S
	Venue type	C	S
	Research purpose	C	I
	Quality attributes	O	S & I
Methods	Methodology	O	S
	Ground truth	O	I
	Results metrics	O	I
	Type of study subjects	O	S & I
	# of study subjects	C	S & I
	Type of truth-set creators	O	S & I
	# Truth-set creators	C	S & I
	Quality of work	C	I
Artefacts,	Granularity of artefact studied	O	I
Activities,	Type of requirements	C	S
and Tools	RE activity	C	S
	Requirements quality tools	O	S

Open (O), Closed (C), Stated (S), Interpreted (I)

**Table 4:** Data extraction attributes: Who, What, Where, and When

(Coding Style)	Description
(Extraction Style)	
**Research purpose** (Closed Coding) (Interpreted)	In the SE literature, there are three main research activities: problem investigation, solution validation, and implementation validation [48]. We map these three phases onto the empirical requirements quality research as articles that 1) seek to *define* requirements quality, 2) propose solutions to *improve* requirements quality, and 3) *evaluate* the research from other articles. As all articles in this SMS must contain empirical evidence (by our inclusion and exclusion criteria), evaluating definitions and improvements proposed in the same article are not labelled as “evaluation”.
**Quality attributes** (Open Coding) (Stated & Interpreted)	The primary purpose of this study is to understand what quality attributes are being addressed within RE research. This open coding category was first extracted as stated. Once the data had been extracted from all papers, thematic analysis was conducted to form codes and themes.

The two possible coding styles are “open” (10/18 questions) and “closed” (8/18). For each closed coding style question, we created the set of possible labels in advance of the data extraction phase. The list of possible labels evolved during the extraction as we learned about the data. Any change in the labels resulted in re-labelling all previously labelled articles.

The three possible extraction styles are “stated” (8/18 questions), “interpreted” (5/18), and “stated & interpreted” (5/18). We extracted “stated” information verbatim from the article; such information must be explicitly written. A primary benefit from this extraction style is that we can clearly map when authors are explicitly *not* stating certain things. We extracted “interpreted” information through analysis of the meaning in the article; this information can be explicitly or implicitly written. Research articles are not written using strict templates, and therefore interpretation is necessary to extract most high-level or novel concepts. To extract “stated & interpreted” information, we first extract them as explicitly written, then we utilised thematic analysis to form high-level interpretations of the stated information. The reason for this combination of techniques is 1) to store and reference the original stated values in the replication package, and 2) to provide value to the reader in presenting our interpretation of the stated information.

We interpreted the data through thematic analysis, thereby producing a set of themes through which the reader can understand the data. The thematic analysis was performed by the first author, with the support of the other authors. Our thematic analysis approach followed the “integrated approach” [49] as we have a mix of both closed and open extraction questions. Our overall approach was more deductive than inductive [49] as even our open extraction questions were grounded regarding the labels, given the specific scope and target of the questions.

**Who, What, Where, and When.** To address RQ1, we created the first data extraction category, “who, what, where, and when”. The extracted questions include the “authors”, “publishing year”, “venue”, and “venue type”. All of this information is stated either in the primary study or online at the publisher’s website. Our SMS had no restriction on the type of venue (conference, journal, workshop, etc.) except that it must be peer-reviewed. The final two extraction questions, “research purpose” and “quality questions” were extracted directly from the primary studies, and required interpretation of the text to be extracted. “Research purpose” will either address the definition, improvement, or evaluation of the quality question(s). “Quality questions” are the specific types of quality being addressed in each article. We list the full details of these extraction questions in Table 4.

**Table 5:** Data extraction attributes: methods

(Coding Style) (Extraction Style)	Description
**Methodology** (Open Coding) (Stated)	The methodology is “the general research strategy that outlines the way in which research is to be undertaken” [50].
**Ground truth** (Open Coding) (Interpreted)	In what way did the researchers align their work with some observable truth? This could be a labelled data set, manual qualitative investigation, etc.
**Results metrics** (Open Coding) (Interpreted)	What metrics did the authors use to describe their results? Precision, recall, f-measure, accuracy, time, etc.
**Type and # of study subjects** (Open Coding) (Stated & Interpreted)	What type of study participants did the researchers study, and how many were there? These participants must have been the subject under study, or tightly coupled with the context of the study such that the results depend on the participants themselves. We extracted this category as stated, and then interpreted it to allow alignment of synonyms used.
**Type and # of truth-set creators** (Open Coding) (Stated & Interpreted)	What type of truth-set creators did the study involve and how many were there? Truth-set creators are used in the creation of truth sets to align the research, empirically, with some observable truth (e.g. the creation of labelled data sets). Truth-set creators are not study subjects because they are not under study; rather, the algorithms are under study. Research where the truth-set creators are also studied (e.g. the labels are studied with respect to the creators) are accounted for as both truth-set creators and study subjects.
**Scientific rigour and industrial relevance** (Open Coding) (Stated & Interpreted)	Ivarsson and Gorschek created a model for evaluating scientific rigour and industrial relevance in technology evaluations [51]. This scheme starts with scoring scientific rigour as 0, 0.5, or 1 across three sub-types: context, study design, and validity. There is then a score for industrial relevance of 0 or 1 across four sub-types: subject, context, scale, and research method.

**Methods.** To address RQ2, we created the second data extraction category, “methods”. The extracted questions begin with the “methodology”, “ground truth”, and “results metrics”. “Methodology” designates the research strategy chosen by the authors in the investigated article to guide their research. “Ground truth” is the technique used by the authors to align their work with some observable truth. “Results metrics” refers to the metric that the authors used to describe their results. The next four questions are related to the participants of the primary studies. These four questions are “type of study subjects”, “# of study subjects”, “type of truth-set creators”, and “# of truth-set creators”. In principle, study subjects have been the subject under study, while truth-set creators are only used for their ability to identify the “truth” in some data set. For both the study subjects and the truth-set creators, we extract the type and number of these subjects used in each article. The final extraction question, “scientific rigour and industrial relevance”, is a scoring system for the scientific rigour and industrial relevance of primary studies across a pre-determined set of criteria [51]. We list the full details of these extraction questions in Table 5.

**Table 6:** Data extraction attributes: artefacts, activities, and tools

(Coding Style) (Extraction Style)	Description
**RE activity** (Closed Coding) (Stated)	The RE phases extracted for this SMS are elicitation, specification, analysis, validation, verification, management, and maintenance [52]. It is common to see “analysis” and “validation” grouped together, as well as “management” and “maintenance” [52]. In this SMS, we separated them so they can be individually labelled depending on how the authors of the primary studies chose to describe their work.
**Granularity of artefact studied** (Open Coding) (Interpreted)	The process of improving the quality of requirements usually involves the input of some artefact to be improved. For this SMS, we are interested in the granularity of these input artefacts: words, sentences, requirements, documents, etc.
**Type of requirements** (Closed Coding) (Stated)	The types of requirements extracted for this SMS are functional and non-functional.
**Requirements Quality Tools** (Open Coding) (Stated)	Which tools were created or used in the primary study? Which quality attributes were addressed by the tool, is there a live link to this tool, what license exists, and who can use it?

**Artefacts, activities, and tools.** To address RQ3, we created the third data extraction, “artefacts, activities, and tools”. The four extracted questions are “granularity of artefact studied”, “type of requirements”, “RE activity”, and “requirements quality tools”. “RE activity” is the stage of the RE process that the research is addressing. “Granularity of artefact studied” is the smallest unit of artefact that the research needs to be performed. “Type of requirements” describes whether the research addresses functional, non-functional, or both types of requirements. “Requirements quality tools” represents which requirements quality tool(s) were created or used in the primary studies. We list the full details of these extraction questions in Table 6.

**Additional extraction questions.** Additional questions were extracted during the data extraction activity, but they are not presented in this SMS. These questions are the title, DOI, method to address, data set used, algorithms applied, results, and contributions. These results can be found in our replication package.

#### Mapping

For the mapping phase, each of the research questions was answered using the extracted data. A process of cleaning and organising was applied to the data, followed by producing a number of figures and tables to visualise the results. The data were analysed and summarised using descriptive statistics and frequency analysis. As a result, there are distinct types of figures displayed: bar charts, stacked bar charts, box plots, and maps (also known as categorical bubble plots). The original data (and the associated codes and themes from the thematic analysis) are available in our replication package. The activity of mapping the data was performed primarily by R1, with close consultation with the other authors.

## Results

### Empirical requirements quality research (RQ1)

#### Who, Where, and When

**Fig. 2:**
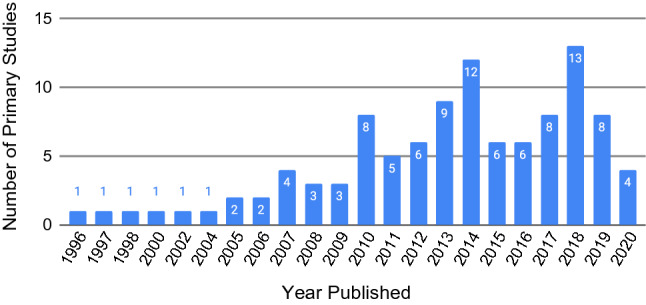
Number of primary studies published per year (N=105)

The primary studies were published across 25 years, from 1996 (January 1) until 2020 (March 27), which is the last day articles were downloaded. With the exception of three years, there was a primary study published every year. There is a general increase over time, with a noticeable increase from 2005 to 2014, followed by a period of highs and lows until 2020. Figure 2 shows the number of publications per year.

**Fig. 3:**
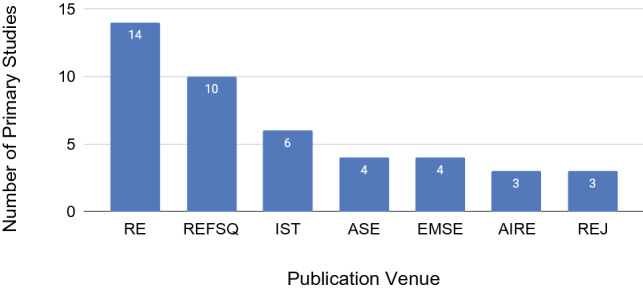
Venues which published 3 or more studies on requirements quality (N=105)

The primary studies were published in 58 different venues. The top six venues with the highest number of published primary studies (three or more), listed in Fig. 3, are Requirements Engineering Conference (RE), Working Conference on Requirement Engineering: Foundation for Software Quality (REFSQ), Information and Software Technology Journal (IST), Conference on Automated Software Engineering (ASE), Empirical Software Engineering Journal (EMSE), Workshop on Artificial Intelligence and Requirements Engineering (AIRE), and the Requirements Engineering Journal (REJ). They account for 42% (44/105) of all primary studies. The number of publications per venue type is 58 at conferences (55%), 38 at journals (36%), and 9 at workshops (9%).

**Fig. 4:**
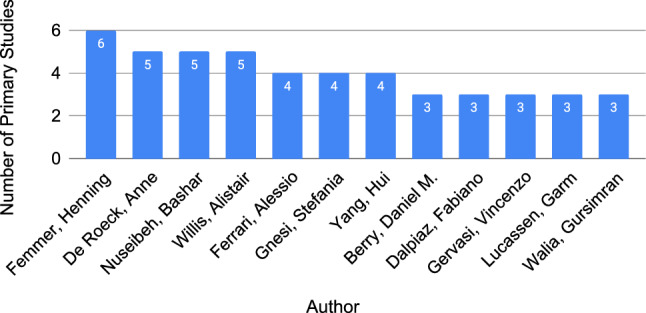
Authors who published 3 or more studies on requirements quality (total unique authors=361)

Finally, the top 12 authors with the highest number of published primary studies (three or more) are shown in Fig. 4. These authors represent 4% (12/280) of the 280 unique authors, but authored 24% (25/105) of the 105 primary studies.

#### Research purpose and quality attributes

**Fig. 5:**
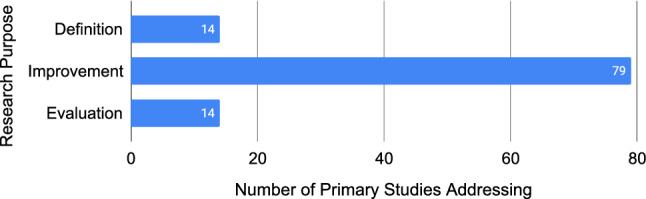
Number of primary studies addressing each research purpose, not mutually exclusive (N=107)

Empirical requirements quality research seeks to *define* or *improve* the quality of requirements, or *evaluate* others’ work on requirements quality. In our sample, 14 primary studies presented a definition, 79 studies proposed a solution for improving requirements quality, and 14 studies performed an evaluation of an existing solution. Figure 5 shows the frequency of each research purpose in the studies (studies can address multiple purposes).

**Fig. 6:**
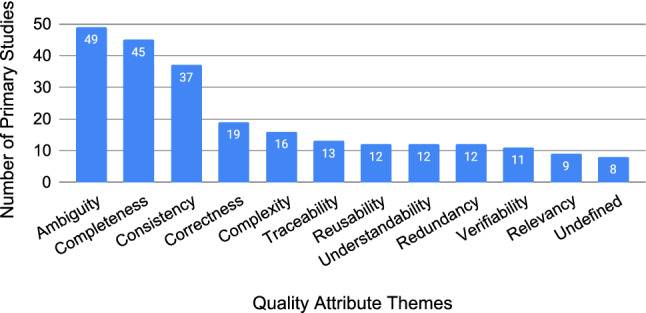
Number of studies addressing each quality attribute, not mutually exclusive (N=243)

Research into requirements quality is characterised by the research goal of addressing one or more quality attributes of requirements. We first extracted the quality attributes as stated in the primary studies, which lead to a list of over 250 unique quality attributes. R1 then performed thematic analysis that lead to a list of codes and themes, which were reviewed and modified through discussion with R2 and R3. Combining similar names and concepts lead to 111 unique quality attribute codes, and grouping those codes lead to 12 quality attribute themes. Figure 6 shows the full breakdown of quality attribute themes and codes. Some quality attribute themes break down into many codes, such as the “ambiguity” theme with 32 codes, where as other themes had no breakdown into codes at all, such as “traceability”. From this point on, we will refer to “quality attribute themes” as “quality attributes”.

**Fig. 7:**
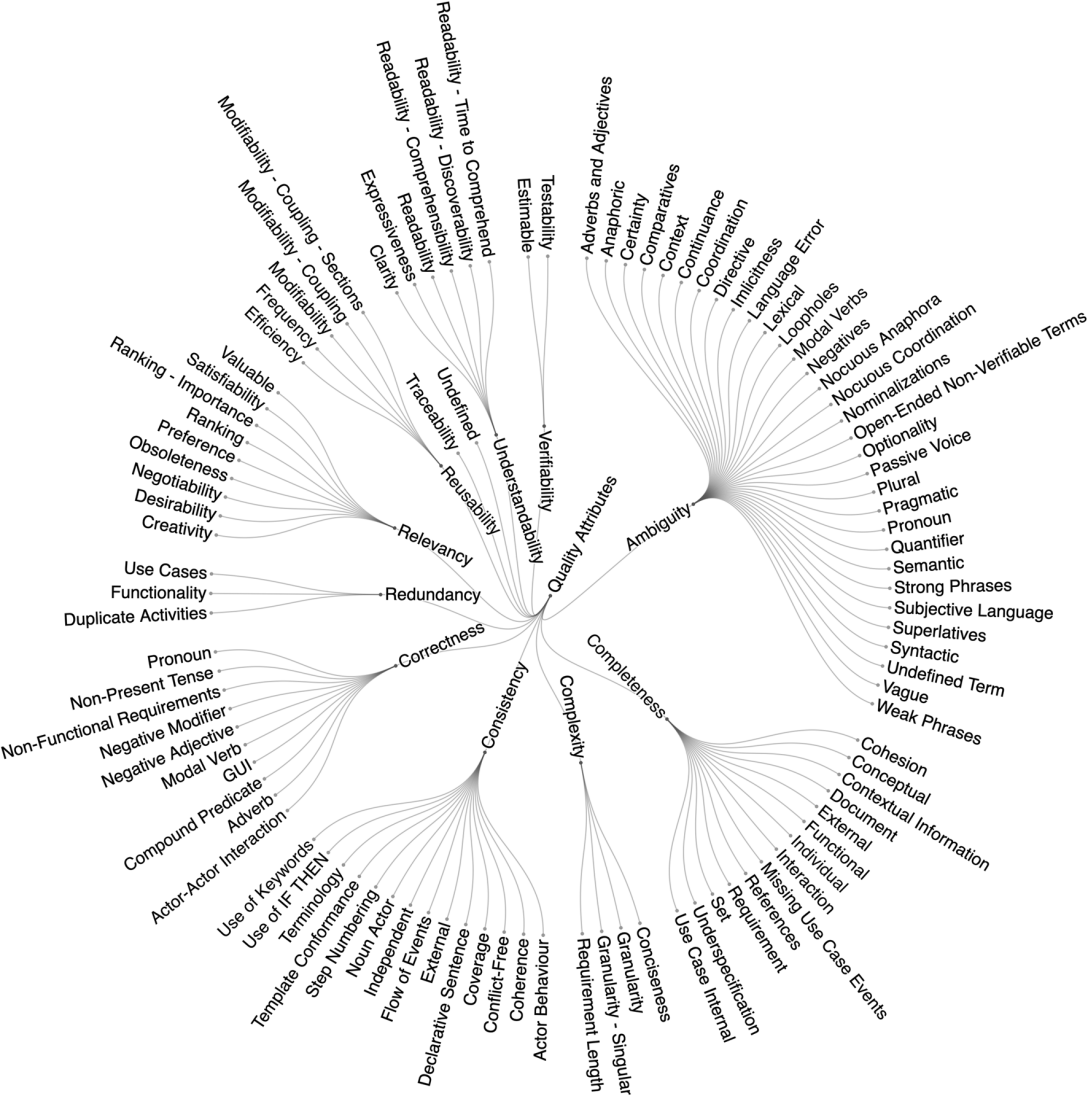
All 111 quality attribute codes from the thematic analysis grouped under the 12 quality attribute themes. Note: if a primary study only refers to a theme (for example, ambiguity) without further specification, then the theme itself is used as code

Figure 7 shows the number of primary studies addressing each of the 12 quality attributes. The quality attributes are not mutually exclusive, and we found that 55% (58/105) of the primary studies address more than one quality attribute. Quality attributes were addressed across the 105 primary studies a total of 243 times. Not captured in Fig. 7 is that some primary studies addressed the same quality attribute more than once—e.g. by working on multiple types of ambiguity. This duplication of themes is not included in our analysis as it unnecessarily complicates the comparison between quality attributes. Full details are available in our replication package.

Figure 7 shows that ambiguity is the most researched quality attribute, representing 20% (49/243) of the researched themes. The first three quality attributes account for 54% (131/243) of all quality attributes addressed. The tail of the graph is fairly consistent at around 12 primary studies addressing each of the remaining themes. “Undefined”, on the far right at 3% (8/243) of themes, represents primary studies that did not specify what kind of quality they were researching. These findings are in alignment with two secondary studies that also found that ambiguity [14], completeness [14, 15], consistency [15], and correctness [14, 15] are the main quality criteria the scientific literature is addressing.

**Fig. 8:**
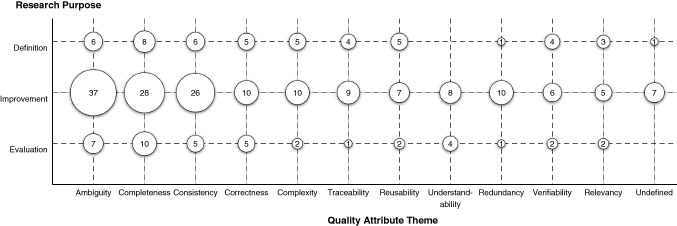
Quality attributes mapped against research purpose for each article

The bubble plot in Fig. 8 shows the interplay of quality attributes and the research purpose of the investigated primary studies. The X and Y axes have categorical values on them, producing a grid-like visualisation. Another dimension, visualised as the size of each bubble, is the number of primary studies where the intersection of the X and Y axes applies. For example, there are 37 studies that have the research purpose of “improvement” and address the quality attribute “ambiguity”.

Comparing Figs. 7 and 5 with the bubble plot in Fig. 8, there is a uniform distribution across both dimensions. For example, the research purpose “improvement” is addressed across all primary studies, as shown in Fig. 5. Additionally, “improvement” is addressed most in each individual quality attribute, without exception, as shown in Fig. 8. Looking at individual data points, there are two notable results. First, the quality attribute “understandability” has zero primary studies addressing the research purpose “definition”. Second, the top three quality attributes (ambiguity, completeness, and consistency) have substantially more primary studies under “improvement” than “definition” or “evaluation”.

### Research methods used (RQ2)

#### Methodologies, ground truth, and results metrics

**Fig. 9:**
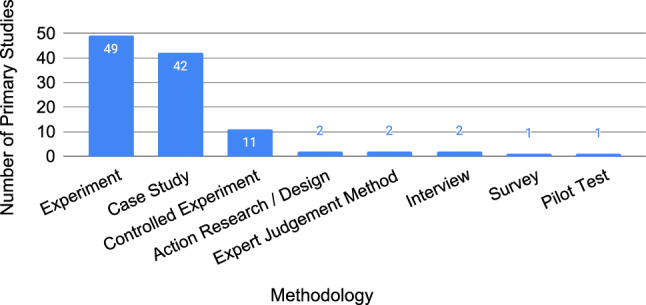
Research methodologies followed in the primary studies, not mutually exclusive (N=110)

Empirical requirements quality research is conducted primarily using experiments and case studies, as shown in Fig. 9, which accounts for 93% (102/110) of methodologies used (studies can use more than one methodology).^11^

**Fig. 10:**
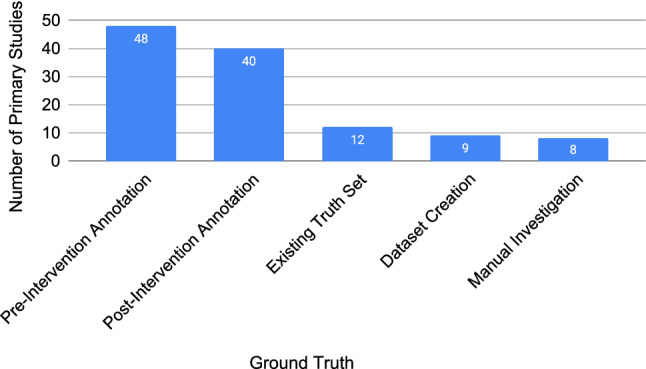
Number of studies and ground truth used, not mutually exclusive (N=117)

In SE research, empiricism is measured in many ways, including changes in data, methods, and algorithms. At the heart of this empiricism, some observable truth must be established. Our primary studies established their ground truths in five different ways (Fig. 10):

- *Pre-intervention annotation*: The data already exist, and the researchers annotate those data before the intervention.
- *Post-intervention annotation*: The data already exist, and the researchers annotate those data after the intervention.
- *Existing truth set*: Utilising an existing truth set from previous work.
- *Data set creation*: Creating the data, explicitly embedding the truth in how it is created. For example, creating use case descriptions with missing steps, where the missing steps are identified by an intervention.
- *Manual investigation*: The data already exist. The researchers investigate the resulting output or consequences following an intervention on that data.

The number of ground truth methods per primary study can be two or more as the studies presented in some articles use multiple methods. Of all ground truth methods used in the primary studies, pre-intervention annotation and post-intervention annotation were used 75% (88/117) of the time. Existing truth set and data set creation appear in 9% (12/117) and 8% (9/117) of the studies, with the final method being manual investigation with 7% (8/117).

**Fig. 11:**
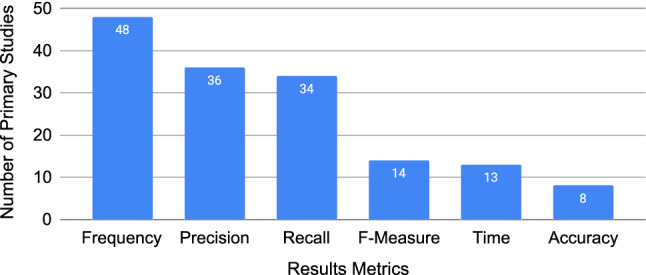
Number of studies per reported result metric; a study can report multiple metrics (N=153)

Figure 11 shows how each primary study reported their results. Across all primary studies, 34% (36/105) used precision, 32% (34/105) recall, and 13% (14/105) used F-measure. Accuracy was used in 8% (8/105) of primary studies; however, it was often used as a general term, without an accompanying definition. 46% (48/105) of the primary studies reported on “frequency”, whether that be of an event, an outcome, a result, etc. Lastly, time was measured and reported in 13 primary studies.

#### Participants

We found that participants in empirical requirements quality research were being used for two distinct reasons: as “study subjects”, but also as “truth-set creators”. Study subjects are participants that were studied by the research, while the truth-set creators are participants tasked with labelling data in order to empirically ground the research results. This distinction is important, because participants can be involved in studies in different ways, therefore influencing threats to validity in different ways. For example, authors of primary studies commonly act as their own truth-set creators, but they should very rarely study themselves as study subjects (except in such cases as action research).

**Fig. 12:**
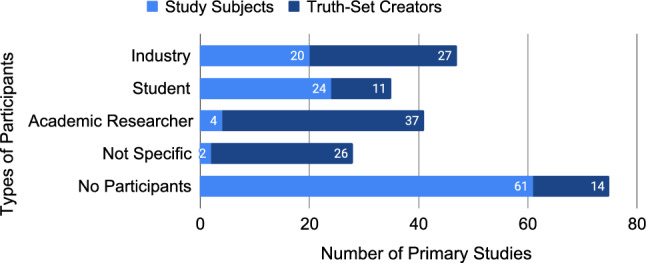
Types of study participants across study subjects and truth-set creators (N=226)

We identified four types of participants, namely industry, student, academic researcher, and not specific. “Not specific” means the authors did not describe their participants clearly, or at all. We also include a special category, “no participants”, when no participants were used in the article. Figure 12 visualises the number of primary studies that utilised study subjects and truth-set creators according to the four types of participants we identified in the primary studies of this SMS. As primary studies can use more than one type of participant, only the “no participants” label is mutually exclusive. Using the “no participants” label, we know that 44 primary studies (105-61) used study subjects (42%) and 91 primary studies (105-14) used truth-set creators (87%). We found that 94% (99/105) of primary studies used at least one type of participant.

**Fig. 13:**
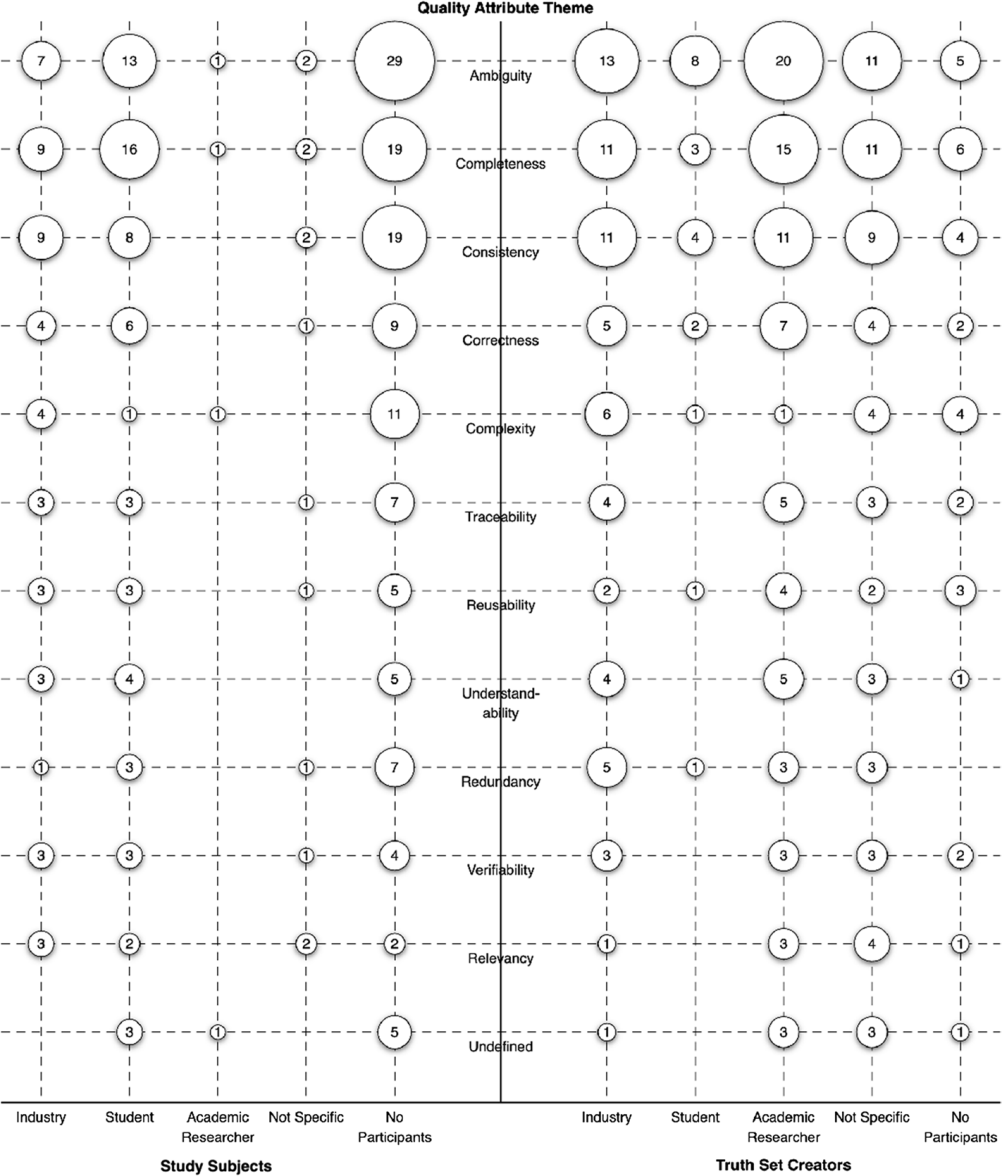
Quality attributes mapped against types of study subjects and truth-set creators

Figure 13 maps the quality attributes against the types of participants. On the left side of the figure, the quality attributes are mapped against study subject types, whereas on the right they are mapped against truth-set creator types. The “no participants” column in both shows the number of primary studies that did not use participants. For example, 29 primary studies investigated ambiguity and did not use study subjects.

**Fig. 14:**
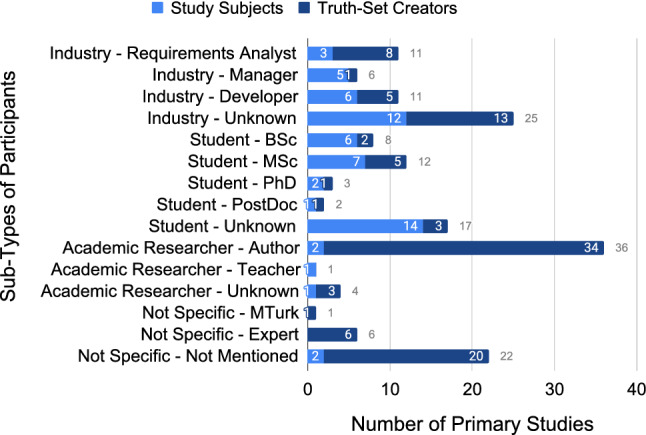
Sub-types of participants across study subjects and truth-set creators (N=165)

Each type of participants (industry, academic, student, and not specific) has a number of sub-types (Fig. 14) that we identified during the extraction phase. Primary studies may utilise more than one participant type or sub-type; we only report on the utilisation per article, not the multiplicity of utilisation per article. For example, if a single primary study utilises “industry - manager” and “industry - developer”, then it is only counted once under Fig. 12 for “industry”, but both are considered in Fig. 14.

Visualising the sub-types of participants highlights additional details in the data. Most importantly, it highlights the “ - Unknown” sub-type for each of the types, which denotes that the authors did not fully describe their participants. For example, for primary studies that reported using industry as participants, 47% (25/53) of them only stated “industry”, with no further details. Details are lacking in 40% (17/42) of the primary studies using students as participants, and 10% (4/41) of primary studies using academic researchers. Included in this idea of “unknown” is reporting participants as “experts”, which has no intrinsic meaning and was used in 7% (6/91) of truth-set creator primary studies. In total, 43% (43/99) of the primary studies describe utilising unknown or expert participants.

Finally, the sub-type “not specific - Not Mentioned” is a label only applied if there was no mention at all of who the study participants were. This accounts for 5% (2/44) of primary studies utilising study subjects, and 22% (20/91) of primary studies utilising truth-set creators. In total, 21% (21/99) of primary studies described utilising participants with no further details.

**Fig. 15:**
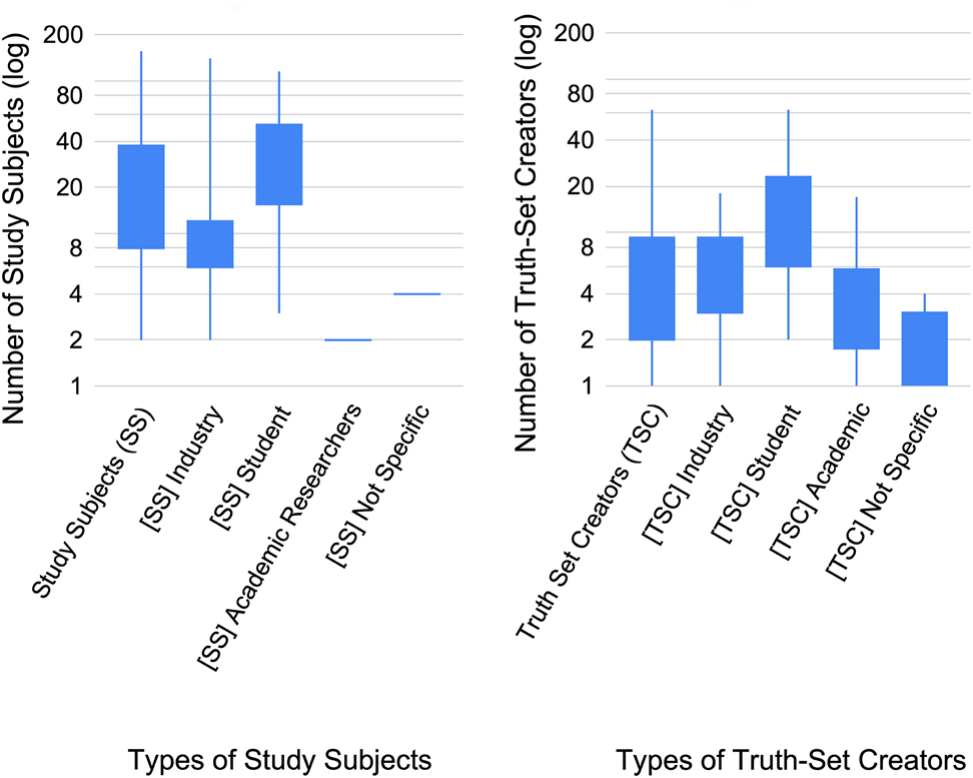
Number of study subjects and truth-set creators, overall and per type, log scale

Figure 15 visualise the number of participants utilised per study subject and truth-set distinction, as well as their respective types. Displayed for each set of data is a box plot with the max, min, first quartile, and third quartile. Note that the y-axis is log-scale to highlight the extremes of both the high and low values. We know that there are 44 primary studies that utilise study subjects and 91 primary studies that utilise truth-set creators; however, not all primary studies that utilise participants report the *number of participants*. Five primary studies that utilise study subjects and 54 primary studies that utilise truth-set creators did not report how many participants were involved. As a result of this missing information, we report the number of participants for 39 study subject primary studies and 37 truth-set creator primary studies.

The first box plot in each figure is the summary of all data across the types, for each of study subjects and truth-set creators. We know from Fig. 12 that double the number of primary studies utilised truth-set creators (91) over study subjects (44). In contrast, we can see from Fig. 15 that the study subject sample sizes are four times larger than truth-set creator sample sizes.^12^

The last four box plots in each of Fig. 15 represent types of participants. Not all data about participants per type can be displayed here due to some primary studies utilising multiple types of participants, and *not distinguishing their respective numbers*. Consequently, during data extraction, we summarised the numbers for each primary study that uses more than one sub-type of participant. As a result, 33% (13/39) of the primary studies with study subjects were unusable, leaving us with 26 primary studies from which we could extract the participants sub-types. For truth-set creators, 19% (7/37) of the primary studies were unusable, leaving 30 primary studies to be analysed for their participants’ sub-types.

The primary result from the sub-types analysis is that students are used in larger groups, much more than industry and academic researchers. This is true for both study subjects and truth-set creators, and more pronounced in the former. For [TSC] student, the first and second quartiles are over 2.5 times higher than industry and academics, and the third quartile is over four times higher.

#### Scientific rigour and industrial relevance

**Fig. 16:**
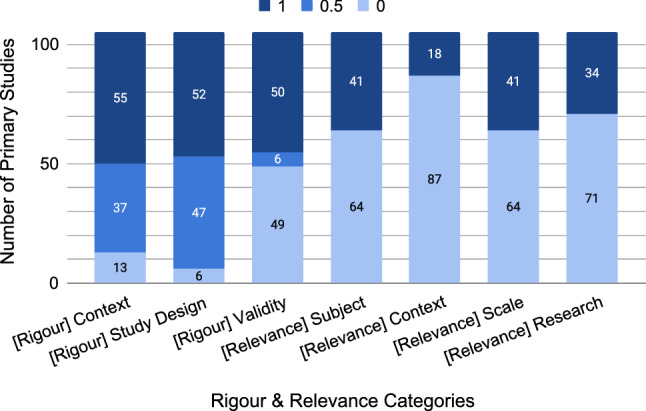
Ratings of scientific rigour and industrial relevance for every article [51] (N=105 for each bar)

Ivarsson and Gorschek [51] outlined a method for evaluating scientific rigour and industrial relevance using a number of criteria (see Table 5). Figure 16 visualises the rigour and relevance values extracted from the 105 primary studies.

The left three bars of Fig. 16 show scores for the scientific “rigour” of the primary studies (either 0, 0.5, or 1). More than half of the primary studies (55) received a score of 1 for [rigour] context, and most of the remaining primary studies received a 0.5, with only 13 primary studies receiving a score of 0. There are similar results for [rigour] study design, which shows an overall good quality when it comes to reporting the scientific rigour of the studies. However, [rigour] validity has almost 50% 0’s, which means 50% of studies did not report any threats to validity.

The right four bars of Fig. 16 show scores for the industrial “relevance” of the primary studies (either 0 or 1). Overall, there is a low industrial involvement in the analysed primary studies. This could be due to a low interest of industrial practice in some of the studied quality aspects, or it could also be due to our exclusion of non-empirical research such as experience reports which can contain industrial relevance. [Relevance] subject and [relevance] scale show the highest values, as each have 41 primary studies that were evaluated as a 1 (39%). [Relevance] research method had 32% (34/105) 1’s over 0’s, and [relevance] context had the lowest overall score with 17% (18/105).

**Fig. 17:**
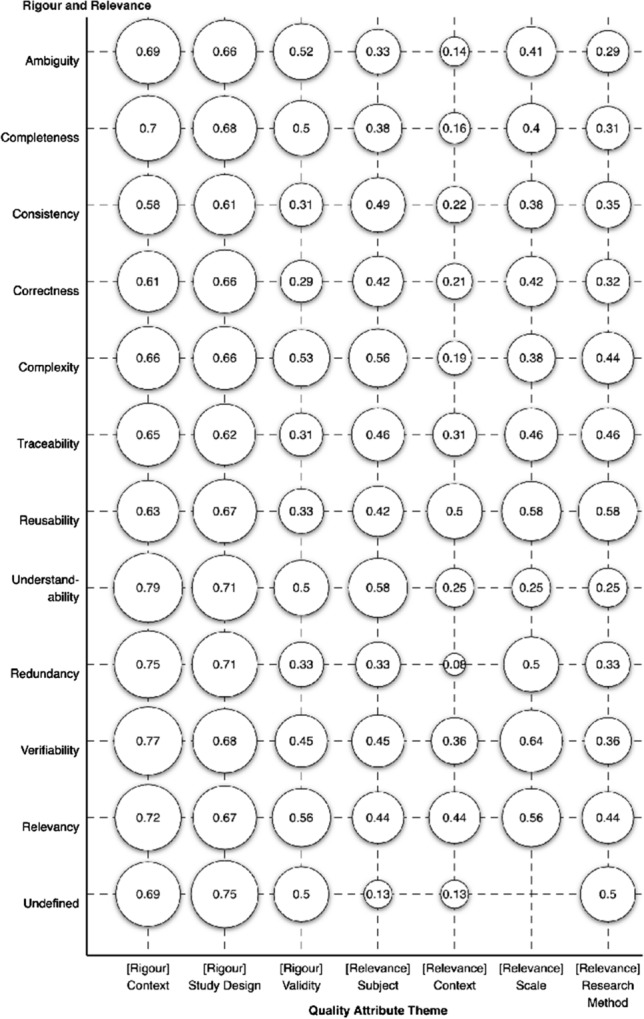
Scientific rigour and industrial relevance mapped against the quality attributes

Figure 17 maps the scientific rigour and industrial relevance values against the quality attributes. The purpose of this map is to provide an in-depth view of how the different quality attributes scored across the different scientific rigour and industrial relevance dimensions. As detailed above, the scientific rigour and industrial relevance extraction category applies ratings of 0, 0.5, or 1 to each of the seven sub-types of quality as per the work of Ivarsson and Gorschek [51]. To translate the distribution of ratings shown in Fig. 6 into single values, we have averaged the ratings for each quality attribute. For example, the quality attribute “understandability” is addressed in 12 primary studies and the ratings given for [rigour] validity are six 1’s and six 0’s, which averages to 0.5 (6/12) as shown in Figure 17.

Looking down the columns of the map (each of the rigour and relevance attributes), there are a few notable findings. All three scientific rigour attributes are fairly consistent across the quality attributes, with [rigour] validity showing some variance in lower values such as consistency, correctness, reusability, traceability, and redundancy. This is similarly true for [relevance] research method which has fairly consistent scores. The remaining industrial relevance sub-types have anomalies, such as [relevance] subject and [relevance] scale that have a very low or zero score for “undefined”. [Relevance] context has the most variance, showing low values for many of the initial and final quality attributes, and mid-tier values for attributes such as reusability, relevancy, verifiability, and traceability.

### Artefacts, activities, and tools (RQ3)

#### RE artefacts

We identified the smallest granularity of RE artefact that is required to perform the research. For example, if an algorithm performs analyses on one sentence at a time, then the type of RE artefact is “sentences”. In another article, if the analysis requires a set of requirements (such as for duplicate requirement detection), then the type of RE artefact is “requirements”. Additionally, we extracted “functional” vs “non-functional” labelling of requirements in the primary studies.

**Fig. 18:**
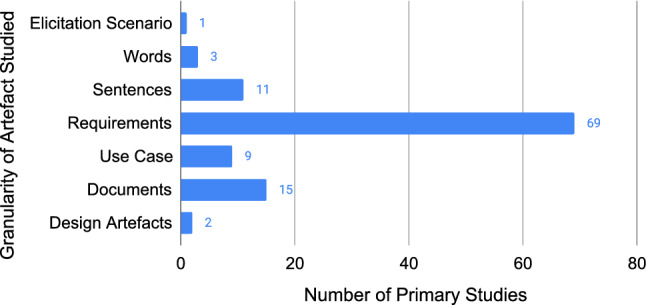
Number of primary studies that used each type of input artefact to their research (N=110)

Figure 18 shows the identified types of RE artefacts, ordered by their evolution through the RE process from elicitation to maintenance^13^. The majority of primary studies, 67% (69/105), utilise the term “requirements”, without being more specific. We found one study that reported on an elicitation scenario,^14^ three that use word-level analyses, and eleven that utilise sentence-level analyses. Looking at the types of requirements artefacts, 9 primary studies focused on use cases, 15 on documents, and 2 on design artefacts. The design artefacts were a system model and mock-ups, both used to communicate perceived requirements captured by system design.

For the types of requirements addressed by each primary study, 32 addressed functional, 3 non-functional, 17 both, and 53 studies did not state the type. Half of the studies did not explicitly state whether the studies they reported dealt with functional or non-functional requirements.

#### RE activities

**Fig. 19:**
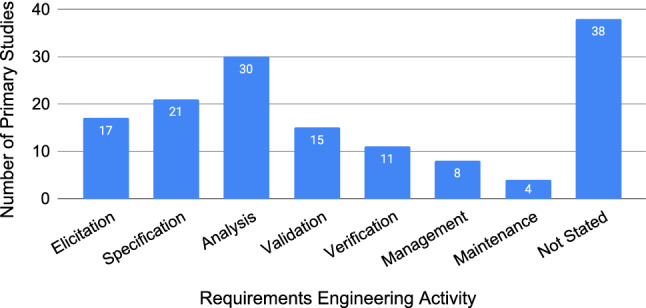
Number of studies addressing each RE activity, not mutually exclusive per article (N=144)

When conducting RE research, a specific RE activity is often targeted. Figure 19 shows the extracted RE activities from primary studies, as stated, from a closed set of labels.

**Fig. 20:**
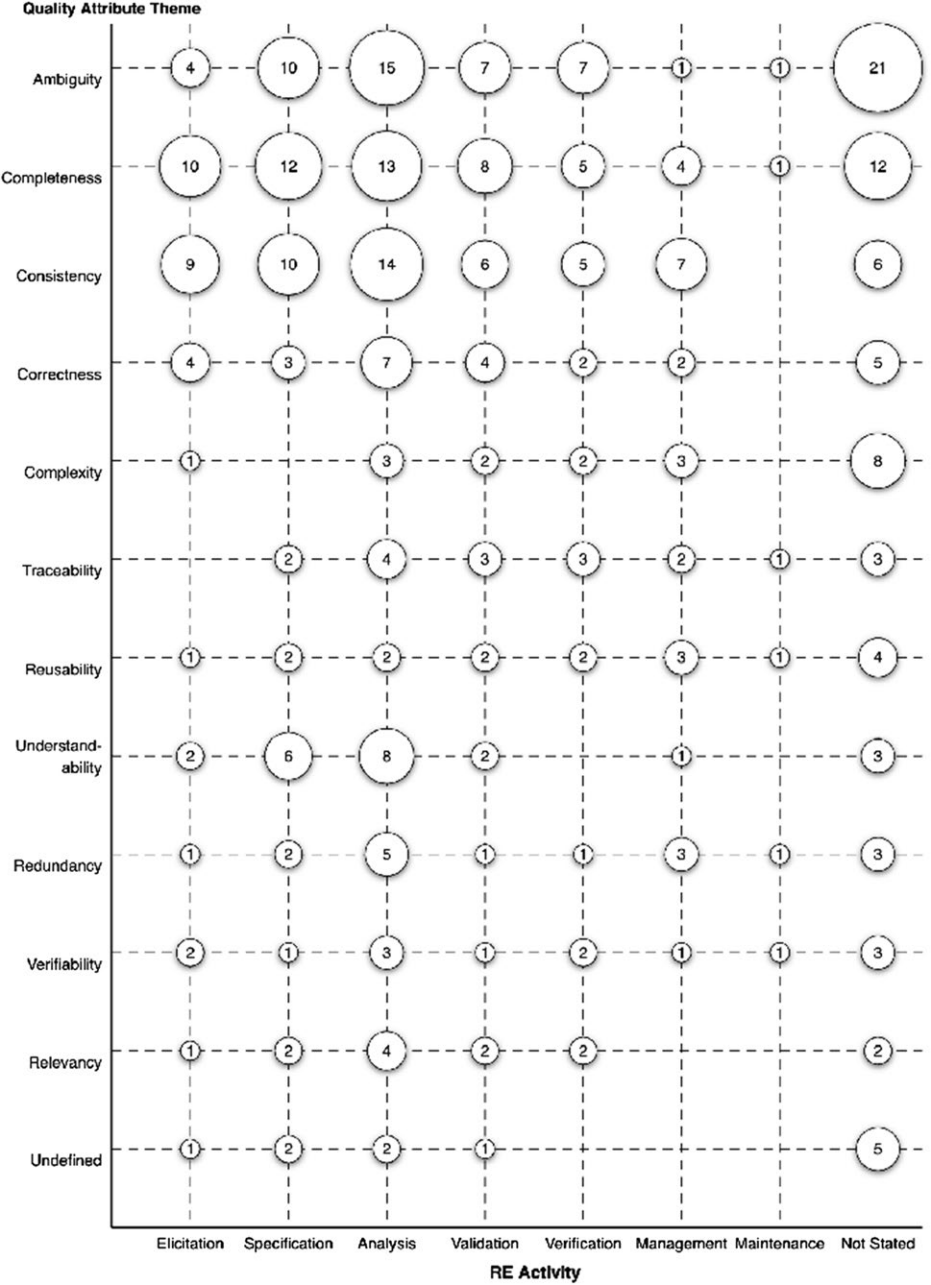
Quality attributes mapped against RE activities

Figure 20 maps the quality attributes against the different RE activities. As the RE activities are ordered chronologically, the bubble plot provides an overview of how the focus on quality attributes is distributed over time (activity). Primary studies about the first four attributes (ambiguity, completeness, consistency, and correctness) are targeting the earlier RE activities, with peak attention on the analysis activity, and very little attention on the maintenance activity. Primary studies about complexity and traceability, in contrast, are more focused on later RE activities. Primary studies about some attributes such as reusability and verifiability are much more evenly spread across the RE activities.

#### Tools

Across the 105 primary studies, 43 created or used a previously created tool for the purpose of requirements quality. In total, we found 41 unique tools as listed in Table 7. For each tool, we list the quality attributes addressed, the primary studies and corresponding years, as well as the link to the tool, the licence, and who can access the tool. Only 15 tools could be found online.

**Fig. 21:**
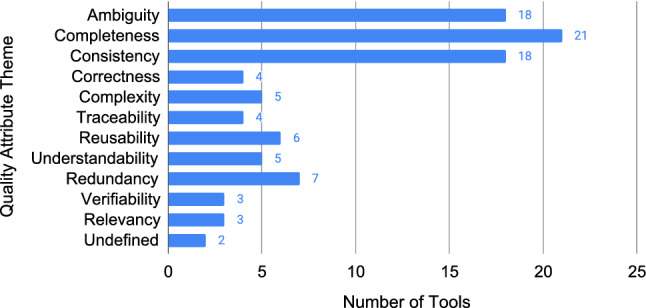
Number of tools addressing each quality attribute; tools can address more than one quality attribute (N=96)

Figure 21 visualises the 41 unique tools according to the quality attribute that they address. Tools can address more than one quality attribute. The attributes in Figure 21 are ranked by their frequency in the primary studies. The top three quality attributes are addressed by a large percentage of the tools: ambiguity at 44% (18/41), completeness at 51% (21/41), and consistency at 44% (18/41). The remaining quality attributes are all addressed about the same number of times, at a median of four times each.

**Table 7:** Requirements quality tools created and utilised

Tool Name	Quality	Year &	Link	LIC	Who
	Attributes	Article			can
	Addressed				Access
ARM	AM CT CY CR	1997 [S42]	https://arm.laplante.io/	–	–
	RL RU TR VB				
TBRIM	AM CX CY	1998 [S7]	–	–	–
QuARS	AM CT CY UB	2005 [S58]	http://quars.isti.cnr.it/	–	–
ElicitO	CY	2008 [S22]	–	–	–
Dowser	AM CT CY	2008 [S36]	http://www.cc.gatech.edu/projects/dowser/	–	–
AIRDoc	CX	2009 [S61]	https://www.cin.ufpe.br/~rar2/airdoc.html	–	–
SQ2E	UD	2010 [S12]	–	–	–
ARBIUM	UD	2010 [S76]	http://www.steam.ualberta.ca/main/research_areas/ARBIUM.htm	–	–
CloneDetective	RD	2010 [S32]	–	–	–
UseCaseAgent	AM CY	2011 [S29]	–		–
OntRep	CY	2011 [S43]	–	–	–
NLARE	AM CT CX	2012 [S46]	–	–	–
		2013 [S47]			
SREE	AM CT	2013 [S50]	–	–	–
		2018 [S39]			
ReqWiki	AM CT TR UB	2013 [S71]	https://github.com/SemanticSoftwareLab/ReqWiki	GNU	All
Reqsec	AM CT CY CR	2013 [S16]	–	–	–
RU TR UB VB					
TextCoop	CT CY	2013 [S41]	–	–	–
CAR	CT	2014 [S4]	–	–	–
IntelliReq	CT CY RD RU	2014 [S15]	–	–	–
DODT	AM CT CY RD	2014 [S78]	–	–	–
RL					
RAT	CT CY UB	2014 [S79]	https://www.bgu.tum.de/era/software/risk-analysis-tool-rat/	–	All
SecMEReq	CT CY	2014 [S19]	–	–-	–
ReUse	RD	2014 [S20]	–	–	–
NLtoSTD-BB	AM CT	2014 [S26]	–	–	–
DeNom	AM	2015 [S53]	–	–	–
ARUgen	AM	2015 [S64]	–	–	–
RETA	CY	2015 [S67]	https://sites.google.com/site/retanlp/	–-	All
Use Case	CT	2015 [S28]	–	–	–
Workbench					
Desiree	AM CT CY RL	2016 [S70]	https://goo.gl/oeJ9Fi	–	All
RU VB					
ReqAligner	RD	2016 [S30]	–	–	–
MaramaAIC	CT CY CR TR	2017 [S60]	–	–	–
Smella	AM CT	2017 [S17]	–	–	–
Randex	CX RU	2017 [S21]	–	–	–
Tactile Check	AM	2017 [S23]	https://github.com/mwmk67/TactileCheck	MIT	All
Grimm Method	AM RD	2017 [S24]	–	–	–
Semios	CY RD	2018 [S99]	https://www.semiosapp.com/en/	–	Paid
REVV	AM CT	2018 [S101]	http://www.staff.science.uu.nl/~dalpi001/revv/	–	–
GATE	CX	2018 [S39]	https://gate.ac.uk/	GNU	All
Traverser	CT	2019 [S83]	http://www.s-lagoon.co.jp/Traverser/	–	–
ReDSeeDS	CR RU UB	2019 [S91]	–	–	–
ScenarioAmigo	CT	2019 [S92]	https://github.com/maniara/ScenarioAmigo	–	–
PASER	CY	2019 [S100]	–	–	–

Quality attribute abbr.: Ambiguity (AM) Completeness (CT)

Complexity (CX) Consistency (CY) Correctness (CR)

Redundancy (RD) Relevancy (RL) Reusability (RU) Verifiability (VB)

Traceability (TR) Undefined (UD) Understandability (UB)

## Discussion

### Empirical requirements quality research (RQ1)

There is a clear preference in empirical requirements quality research to offer improvements over definitions or evaluations (Fig. 5). While the logical research progression for any topic would be to define, then improve, and then evaluate, empirical requirements quality research has largely focused on improvements. It could be argued that this research field has reached saturation of definitions for each quality attribute and therefore has no need for further definitions. However, Fig. 8 only shows the 12 quality attribute *themes*, of which there are 111 unique quality attribute *codes* (see Fig. 6). For example, the quality attribute “ambiguity” has 32 unique types of ambiguity, and only six empirical definitions. Each of those quality attribute codes is likely a complex research topic that should be understood empirically. One reason for such a lack of definitions could be our exclusion of standards, books, and non-empirical research papers; however, our goal was to map requirements quality as understood empirically to present an evidence-based perspective. We believe future requirements quality research should focus on empirically understanding and defining the quality concepts being addressed. This could be done, for example, through interview studies with practitioners or analysing legacy requirements documents to characterise various quality attributes.

There is a preference in empirical requirements quality research to investigate ambiguity, completeness, and consistency, with correctness not far behind (Fig. 7). This could be due to the IEEE standards [8, 9, 13] in which these attributes are listed first^15^. This could also be due to the concept of “The Three Cs of Requirements” in which Zowghi and Gervasi argue that “there is an important causal relationship between consistency, completeness and correctness” [53]. The IEEE 29148-2011 and -2018 standards each have a separate section dedicated to “requirement language criteria” which lists “types of unbounded or ambiguous terms”, potentially explaining why ambiguity has received such attention. From a requirements perspective, ambiguity could be interpreted as in direct conflict with the concept of documenting the formal needs for a system. This has largely been the motivating argument for primary studies that investigate ambiguity (e.g. [54–58]). However, there is also a discussion around the potential importance of ambiguity in requirements, particularly in the early activities of RE, where ambiguity could be “a powerful tool to discover tacit knowledge during requirements elicitation” [59]. Overall, there is a preference for researching these four primary quality attributes, and future research is needed on the others.

### Research methods used (RQ2)

We observed a clear preference in empirical requirements quality research for case studies and experiments (including controlled experiments), as shown in Fig. 9 where these methodologies account for 93% (102/110) of all methodologies applied. A secondary study by Höfer and Tichy in 2007 also found that experiments and case studies were the top used research methods in empirical software engineering (SE) research: 37.6% of the primary studies report experiments, 28.6% case studies, and 33.8% other methods [60]. Our findings show a less diverse use of research methods in empirical requirements quality research, with 54.5% experiments, 38.2% case studies, and 7.3% other methods—i.e. a drop of 26 percentage points in “other” methods compared to Höfer and Tichy [60]. Empirical requirements quality research would benefit from more diversity given that each method has unique benefits and trade-offs [61].

We also found a clear preference of pre-intervention annotation and post-intervention annotation as sources of truth (73% in total as shown in Fig. 10). This manual work is often necessary, but over time should lead to shared truth sets in the community. Future empirical requirements quality research should aim to reuse truth sets to reduce the research workload, and align the research being conducted with comparable results.

There is also a clear distinction between study subjects and truth-set creators. Such distinction is from our interpretation of the reported research, and not explicitly described in the primary studies. We see a benefit in understanding the differences, selecting the appropriate type, utilising those differences in the research, and reporting them explicitly. Another finding from our investigation of participants in empirical requirements quality research is that many primary studies do not describe who their participants are in enough detail 43% (43/99), or at all 21% (21/99). The use of participants is necessary to validate research, but importantly, who the participants are and why they are being utilised is key to that validation. Our results show a deficiency in the reporting of participants, and this should be addressed in future empirical requirements quality research.

Our results suggest that empirical requirements quality research has fairly good scientific rigour, but low industrial relevance (Fig. 16). [Rigour] context and [rigour] study design have the strongest ratings at 70% for context and 72% for study design. Notably, half of the primary studies have a 1, and very few primary studies have a 0, which shows a high commitment to detailing the context and study design. Scientific rigour suffers the most from a lack of reporting the threats to validity. Figure 16 shows that for validity, primary studies either describe it in full, or do not mention it at all. Industrial relevance has the most room for improvement. Most notably, only 18 out of 105 primary studies report on research validated in an industrial context. Additionally, cross-checking the extracted data shows that half of those 18 primary studies did not use study subjects, meaning that they only utilised the industrial context for the labelling of data. Accordingly, only 9% (9/105) of primary studies applied their work in context. As described by Nuseibeh and Easterbrook: “modelling and analysis cannot be performed adequately in isolation from the organisational and social context in which any new system will have to operate” [62]. There is a need for empirically grounded requirements quality research in context, particularly in the industrial context.

### Artefacts, activities, and tools (RQ3)

Empirical requirements quality research is primarily targeting the analysis activity, with a gradual drop off in either direction as visualised in Fig. 19. The mapping of RE activities against quality attributes in Fig. 20 reveals granular insights into these activities. Analysis, for example, shows an increased number of primary studies for the last four of five quality attributes. There is a deficiency of research in the management and maintenance activities. Overall, there is room for researchers to investigate RE activities more evenly, focusing their efforts on the initial and final activities of the RE process.

A majority of empirical research into requirements quality (67%) focused on the generic idea of “requirements”, without considering other more specific forms. This gap shows much room for novel research on elicitation scenarios, use cases, and user stories, among many other types of requirements. The fact that we do not see more non-textual requirements types in this study might be because there has not been an explicit effort to improve the quality of those types of requirements. Alternatively, it could be because researchers working on, for example, requirements models did not explicitly use the term “quality” in their studies.^16^ Future empirical requirements quality research should address a more diverse granularity of requirements artefacts, including alternative forms of requirements such as use cases, user stories, or feature requests in issue trackers.

As for functional vs non-functional, it appears that the preference is to research functional aspects of requirements over non-functional. This is likely due to the specificity of functional requirements, which opens them up to greater scrutiny when considering their quality. We recommend future research takes a closer look at non-functional requirements and how quality can be defined, improved, and evaluated.

The RE research community developed 41 requirements quality tools (15 of which are available online), which is a good sign of solution-based research. However, the majority of these tools are created new, and never used again. There is a strong need for empirical requirements quality research to publish archived versions of tools for future researchers to build on. The majority of requirements quality techniques being developed and researched do not need to develop their own tools from the ground up. An example of this is how researchers commonly visualise their quality recommendations as annotations: this could exist as a standard tool that requirements quality researchers use to visualise their output.

With respect to quality attributes, a majority of the tools cover ambiguity, completeness, and consistency. For researchers looking to conduct empirical requirements quality research into these quality attributes, it is highly recommended to look into existing tools first.

## Study validity

### Research reliability

Reliability is defined as the degree to which different researchers, following our methodology, would produce similar results [63]. We take inspiration from Zhao et al. [16] in structuring our reliability section by primary phases of our SMS.

Ensuring the reliability of the article search is important for gathering *all* relevant articles. We followed the recommendations provided by Petersen [35] and Kitchenham [38] in designing the search phase, as well as in selecting the sources [16, 37, 64]. The primary study search phase consists of applying the search on databases, duplicate removal, and title skimming. For our database search, we used the indexing services Google Scholar and Web of Science. To increase reliability in our results, we also collected articles from the primary publisher databases of RE-related research (IEEE, ACM, Elsevier, and Springer) [16]. By employing the most common search strategy in systematic reviews in SE [35], we believe we mitigated any significant selection biases in the search strategy.

The reliability of the article selection phase is important to prevent false-negatives and avoid removing important articles. For the candidate selection activity, the articles were independently peer-labelled, and disagreements were carefully discussed according to the guidelines of Petersen at al. [35]. If there was uncertainty towards an article after discussion, the default decision was to include it, thereby mitigating the threat of false-negatives. The title selection, abstract selection, and PDF selection activities were performed by R1 alone due to the simplicity of the task. While the above three activities are grouped together with the candidate selection activity in Fig. 1, they are completely different tasks. The aforementioned three activities were a matter of visually identifying clear issues, whereas the candidate selection activity was a matter of academic inquiry and discussion. To increase reliability of these solo tasks, *any* uncertainty towards the inclusion of an article leads to it being included and therefore being kept for an upcoming peer-involved task.

The reliability of the data extraction phase is important to ensure the correctness of the extracted questions. The first four authors—without any hierarchy among them—carefully conducted the data extraction phase, with two authors independently doing a full-read of each article. Following the extraction, the researchers met to discuss and agree on the extracted questions. The fifth author oversaw the process and was included in the discussion in case of an ambiguous issue. We performed open-coding and thematic analysis on a few extraction questions, as described in Sect 3.2.3. The original data and labels are available in our replication package.

The reliability of the mapping phase is important to ensure that the interpretation of the data is objective and aligned with the original intent behind the extracted data. All five authors were involved with the interpretation of the data, and transformation of the raw data to be reported in the form of descriptive statistics, tables, and figures. This checking between authors leads to several changes in how the data were transformed and interpreted based on internal feedback rounds. Additionally, the original data, tables, and figures are available in our replication package.

### Threats to validity

*Construct validity* The primary threat to construct validity is in the data extraction phase. Agreement among the authors was reached before beginning the full extraction run, and a trial extraction run was conducted for each researcher involved in this activity to align their understandings of the questions to extract. This effort resulted in changing extraction question names, descriptions, coding styles, and extraction styles to meet the understanding of the researchers. Thereafter, the process of extracting the questions involved very few changes during the full run.

*Internal validity* The design of this SMS follows the recommendations of standard articles outlining how to conduct an SMS in SE research, and we carefully followed those recommendations. The data presented are available in our replication package to be checked by anyone interested, increasing the likelihood that critical errors in reporting (if any) can and will be caught.

*External validity* Systematic studies are designed to be representative across the dimensions of the research area being investigated. While we are unable to fully exclude the potential threat of possibly missing primary studies, we have done our best to ensure the rigour of our work and thus feel confident about the generalisability of our claims in the specific research area of empirical research on requirements quality. When applying our results beyond this specific scope, caution must be used. While sub-communities can be representative of their broader community, this claim cannot be made without evidence sampled specifically from that broader community. We leave it to tertiary research to take our claims about requirements quality and look for similar patterns in the broader RE—and potentially SE—communities.

## Conclusions

This SMS offers an overview of the field of empirical research on requirements quality. We analysed the quality attributes researched in previous published work, the research methods used, and the studied RE activities, artefacts, and tools. Our detailed reporting and replication package can be used to understand cross sections of the data.

We found a broad set of researchers publishing at specific venues such as RE, REFSQ, and REJ. The majority of primary studies focus on researching improvements to requirements quality, with very few focusing on definitions or evaluations. Most frequently studied requirements quality attributes are ambiguity, completeness, consistency, and correctness.

We found that empirical requirements quality researchers primarily use experiments and case studies. For participants, we found a distinction between study subjects and truth-set creators. More studies utilised industry participants than students; however, the *number of participants* in student studies was three times higher than industry studies. We also observed an issue with the number of primary studies reporting incomplete information about their participants. Finally, the scientific rigour and industrial relevance analysis revealed that rigour is quite good (with the exception of validity), but relevance is low.

As for the particular RE focus, we found that empirical requirements quality research has primarily focused on “requirements”, with little attention given to other artefacts such as use cases, user stories or issues in issue trackers. The focus was also on the specification and analysis activities, with less attention on elicitation, validation, and verification, and very little attention to management and maintenance. Finally, we found 41 unique requirements quality tools, many of which contain no reference on how to find them and only three of them contained a visible license.

Our thorough results and detailed discussion serve as a starting point for future empirical requirements quality researchers. We hope that future research will take our insights into consideration as this field continues to grow.

**Supplementary information** All supplementary information can be found in our replication package^17^

## Appendix A: Primary studies

1. Lombriser P, Dalpiaz F, Lucassen G, Brinkkemper S. Gamified requirements engineering: model and experimentation. In International Working Conference on Requirements Engineering: Foundation for Software Quality 2016 Mar 14 (pp. 171-187). Springer, Cham.
2. Tjong SF, Berry DM. The design of SREE—a prototype potential ambiguity finder for requirements specifications and lessons learned. In International Working Conference on Requirements Engineering: Foundation for Software Quality 2013 Apr 8 (pp. 80-95). Springer, Berlin.
3. Ferrari A, Gnesi S, Tolomei G. Using clustering to improve the structure of natural language requirements documents. In International Working Conference on Requirements Engineering: Foundation for Software Quality 2013 Apr 8 (pp. 34-49). Springer, Berlin.
4. Ferrari A, Dell’Orletta F, Spagnolo GO, Gnesi S. Measuring and improving the completeness of natural language requirements. In International Working Conference on Requirements Engineering: Foundation for Software Quality 2014 Apr 7 (pp. 23-38). Springer, Cham.
5. Femmer H, Unterkalmsteiner M, Gorschek T. Which requirements artifact quality defects are automatically detectable? A case study. In2017 IEEE 25th International Requirements Engineering Conference Workshops (REW) 2017 Sep 4 (pp. 400-406). IEEE.
6. Femmer H, Mund J, Méndez D. It’s the activities, stupid! a new perspective on RE quality. In 2015 IEEE/ACM 2nd International Workshop on Requirements Engineering and Testing 2015 May 18 (pp. 13-19). IEEE.
7. Romano JJ, Palmer JD. TBRIM: decision support for validation/verification of requirements. In IEEE International Conference on Systems, Man, and Cybernetics 1998 Oct 14 (Vol. 3, pp. 2489-2494). IEEE.
8. Ogata S, Aoki Y, Okuda H, Matsuura S. An Automation of Check Focusing on CRUD for Requirements Analysis Model in UML. International Journal of Computer, Electrical, Automation, Control and Information Engineering. 2012 Sep 1;6(9):1149-5.
9. Phalp KT, Vincent J, Cox K. Assessing the quality of use case descriptions. Software Quality Journal. 2007 Mar;15(1):69-97.
10. Aranda GN, Vizcaíno A, Piattini M. A framework to improve communication during the requirements elicitation process in GSD projects. Requirements Engineering. 2010 Nov 1;15(4):397-417.
11. Walia GS, Carver JC. Using error abstraction and classification to improve requirement quality: conclusions from a family of four empirical studies. Empirical Software Engineering. 2013 Aug;18(4):625-58.
12. Aceituna D, Do H, Lee SW. SQ2E: An approach to requirements validation with scenario question. In 2010 Asia Pacific Software Engineering Conference 2010 Nov 30 (pp. 33-42). IEEE.
13. Osada A, Ozawa D, Kaiya H, Kaijiri K. The role of domain knowledge representation in requirements elicitation. In 25th IASTED International Multi-Conference Software Engineering 2007 Feb 13 (pp. 84-92).
14. Park S, Kim H, Ko Y, Seo J. Implementation of an efficient requirements-analysis supporting system using similarity measure techniques. Information and Software Technology. 2000 Apr 15;42(6):429-38.
15. Ninaus G, Reinfrank F, Stettinger M, Felfernig A. Content-based recommendation techniques for requirements engineering. In 2014 IEEE 1st International Workshop on Artificial Intelligence for Requirements Engineering (AIRE) 2014 Aug 26 (pp. 27-34). IEEE.
16. Daramola O, Sindre G, Moser T. A tool-based semantic framework for security requirements specification. Journal of Universal Computer Science. 2013;19(13):1940-62.
17. Femmer H, Méndez D, Wagner S, Eder S. Rapid quality assurance with requirements smells. Journal of Systems and Software. 2017 Jan 1;123:190-213.
18. Issa AA, AlAli AI. Automated requirements engineering: use case patterns-driven approach. IET Software. 2011 Jun 9;5(3):287-303.
19. Yahya S, Kamalrudin M, Sidek S, Grundy J. Capturing security requirements using essential use cases (EUCs). In Requirements Engineering 2014 (pp. 16-30). Springer, Berlin.
20. Rago AM, Frade P, Ruival M, Marcos C. An Approach for Automating Use Case Refactoring. Sociedad Argentina de Informatica E Investigacion Operativa, p. 15, 2014.
21. Antinyan V, Staron M. Rendex: A method for automated reviews of textual requirements. Journal of Systems and Software. 2017 Sep 1;131:63-77.
22. Al Balushi T, Sampaio PR, Patel M, Corcho O, Loucopoulos P. Identifying NFRs conflicts using quality ontologies. 20th International Conference on Software Engineering and Knowledge Engineering SEKE 2008 Jul;929-934.
23. Wilmink M, Bockisch C. On the ability of lightweight checks to detect ambiguity in requirements documentation. In International Working Conference on Requirements Engineering: Foundation for Software Quality 2017 Feb 27 (pp. 327-343). Springer, Cham.
24. Lucassen G, Dalpiaz F, van der Werf JM, Brinkkemper S. Improving user story practice with the Grimm Method: A multiple case study in the software industry. In International Working Conference on Requirements Engineering: Foundation for Software Quality 2017 Feb 27 (pp. 235-252). Springer, Cham.
25. Parra E, Dimou C, Llorens J, Moreno V, Fraga A. A methodology for the classification of quality of requirements using machine learning techniques. Information and Software Technology. 2015 Nov 1;67:180-95.
26. Aceituna D, Walia G, Do H, Lee SW. Model-based requirements verification method: Conclusions from two controlled experiments. Information and Software Technology. 2014 Mar 1;56(3):321-34.
27. Femmer H, Kučera J, Vetrò A. On the impact of passive voice requirements on domain modelling. In 2014 8th ACM/IEEE International Symposium on Empirical Software Engineering and Measurement Sep 18 (pp. 1-4).
28. Jurkiewicz J, Nawrocki J. Automated events identification in use cases. Information and Software Technology. 2015 Feb 1;58:110-22.
29. Massollar JL, de Mello RM, Travassos GH. Investigating the Feasibility of a Specification and Quality Assessment Approach Suitable for Web Functional Requirements. In 2011 International Conference of the Chilean Computer Science Society 2011 Nov 9 (pp. 108-117). IEEE.
30. Rago A, Marcos C, Diaz-Pace JA. Identifying duplicate functionality in textual use cases by aligning semantic actions. Software & Systems Modeling. 2016 May 1;15(2):579-603.
31. Kaiya H, Saeki M. Using domain ontology as domain knowledge for requirements elicitation. In 14th IEEE International Requirements Engineering Conference (RE’06) 2006 Sep 11 (pp. 189-198). IEEE.
32. Juergens E, Deissenboeck F, Feilkas M, Hummel B, Schaetz B, Wagner S, Domann C, Streit J. Can clone detection support quality assessments of requirements specifications?. In Proceedings of the 32nd ACM/IEEE International Conference on Software Engineering-Volume 2 2010 May 1 (pp. 79-88).
33. Kabeli J, Shoval P. Comprehension and quality of analysis specification—a comparison of FOOM and OPM methodologies. Information and Software Technology. 2005 Mar 15;47(4):271-90.
34. Körner SJ, Landhäußer M, Tichy WF. Transferring research into the real world: How to improve RE with AI in the automotive industry. In 2014 1st International Workshop on Artificial Intelligence for Requirements Engineering (AIRE) 2014 Aug 26 (pp. 13-18). IEEE.
35. Din CY, Rine DC. Requirements content goodness and complexity measurement based on NP chunks. Journal of Systemics, Cybernetics and Informatics. VDM Publishing; 2008.
36. Popescu D, Rugaber S, Medvidovic N, Berry DM. Reducing ambiguities in requirements specifications via automatically created object-oriented models. In 2007 Monterey Workshop Sep 10 (pp. 103-124). Springer, Berlin.
37. och Dag JN, Regnell B, Carlshamre P, Andersson M, Karlsson J. A feasibility study of automated natural language requirements analysis in market-driven development. Requirements Engineering. 2002 Apr 1;7(1):20-33.
38. Dorigan JA, de Barros RM. Requirements Engineering: A Process Model and Case Study to Promote Standardization and Quality Increase. 7th International Conference on Software Engineering Advances ICSEA 2012.
39. Ferrari A, Gori G, Rosadini B, Trotta I, Bacherini S, Fantechi A, Gnesi S. Detecting requirements defects with NLP patterns: an industrial experience in the railway domain. Empirical Software Engineering. 2018 Dec;23(6):3684-733.
40. Nistala PV, Nori KV, Natarajan S. Process patterns for requirement consistency analysis. In 21st European Conference on Pattern Languages of Programs 2016 Jul 6 (pp. 1-11).
41. Kang J, Saint-Dizier P. Discourse structure analysis for requirement mining. International journal of Knowledge Content Development & Technology. 2013;3(2):43-65.
42. Wilson WM, Rosenberg LH, Hyatt LE. Automated analysis of requirement specifications. In 19th International Conference on Software Engineering 1997 May 1 (pp. 161-171).
43. Moser T, Winkler D, Heindl M, Biffl S. Automating the detection of complex semantic conflicts between software requirements. In 23rd International Conference on Software Engineering and Knowledge Engineering, Miami 2011.
44. Dzung DV, Ohnishi A. Evaluation of ontology-based checking of software requirements specification. In 2013 IEEE 37th Annual Computer Software and Applications Conference 2013 Jul 22 (pp. 425-430). IEEE.
45. Yang H, De Roeck A, Gervasi V, Willis A, Nuseibeh B. Analysing anaphoric ambiguity in natural language requirements. Requirements engineering. 2011 Sep 1;16(3):163.
46. Huertas C, Juárez-Ramírez R. NLARE, a natural language processing tool for automatic requirements evaluation. In Proceedings of the CUBE International Information Technology Conference 2012 Sep 3 (pp. 371-378).
47. Huertas C, Juárez-Ramírez R. Towards assessing the quality of functional requirements using English/Spanish controlled languages and context free grammar. In Proc. Third International Conference on Digital Information and Communication Technology and its Applications (DICTAP 2013), Ostrava, Czech Republic on 2013 Jul 8 (pp. 234-241).
48. Ott D. Defects in natural language requirement specifications at mercedes-benz: An investigation using a combination of legacy data and expert opinion. In 2012 20th IEEE International Requirements Engineering Conference (RE) 2012 Sep 24 (pp. 291-296). IEEE.
49. Yang H, Willis A, De Roeck A, Nuseibeh B. Automatic detection of nocuous coordination ambiguities in natural language requirements. In Proceedings of the IEEE/ACM International Conference on Automated Software Engineering 2010 Sep 20 (pp. 53-62).
50. Gleich B, Creighton O, Kof L. Ambiguity detection: Towards a tool explaining ambiguity sources. In International Working Conference on Requirements Engineering: Foundation for Software Quality 2010 Jun 30 (pp. 218-232). Springer, Berlin.
51. Sinpang JS, Sulaiman S, Idris N. Detecting Ambiguity in Requirements Analysis Using Mamdani Fuzzy Inference. Journal of Telecommunication, Electronic and Computer Engineering (JTEC). 2017 Oct 20;9(3-4):157-62.
52. Bäumer FS, Geierhos M. Flexible ambiguity resolution and incompleteness detection in requirements descriptions via an indicator-based configuration of text analysis pipelines. In: Proceedings of the 51st Hawaii International Conference on System Sciences, 2018 (pp. 5746–5755).
53. Landhaußer M, Korner SJ, Tichy WF, Keim J, Krisch J. DeNom: a tool to find problematic nominalizations using NLP. In 2015 IEEE Second International Workshop on Artificial Intelligence for Requirements Engineering (AIRE) 2015 Aug 24 (pp. 1-8). IEEE.
54. Dorigan JA, de Barros RM. A model of requirements engineering process for standardization and quality increase. In 2012 IADIS International Conference Applied Computing (pp. 343-347).
55. Eckhardt J, Vogelsang A, Femmer H, Mager P. Challenging incompleteness of performance requirements by sentence patterns. In 2016 IEEE 24th International Requirements Engineering Conference (RE) 2016 Sep 12 (pp. 46-55). IEEE.
56. Christophe F, Mokammel F, Coatanéa E, Nguyen A, Bakhouya M, Bernard A. A methodology supporting syntactic, lexical and semantic clarification of requirements in systems engineering. International Journal of Product Development. 2014 Jan 1;19(4):173-90.
57. Nikora A, Hayes J, Holbrook E. Experiments in automated identification of ambiguous natural-language requirements. In 2010 IEEE International Symposium on Software Reliability Engineering, San Jose: IEEE Computer Society.
58. Lami G, Ferguson R, Goldenson D, Fusani M, Fabbrini F, Gnesi S. 2.4. 2 QuARS: Automated Natural Language Analysis of Requirements and Specifications. In INCOSE International Symposium 2005 Jul (Vol. 15, No. 1, pp. 344-353).
59. Port D, Nikora A, Hayes JH, Huang L. Text mining support for software requirements: Traceability assurance. In 2011 44th Hawaii International Conference on System Sciences 2011 Jan 4 (pp. 1-11). IEEE.
60. Kamalrudin M, Hosking J, Grundy J. MaramaAIC: tool support for consistency management and validation of requirements. Automated Software Engineering. 2017 Mar 1;24(1):1-45.
61. Ramos R, Castro J, Alencar F, Araújo J, Moreira A, da Computacao CD, Penteado R. Quality improvement for use case model. In 2009 XXIII Brazilian Symposium on Software Engineering 2009 Oct 5 (pp. 187-195). IEEE.
62. España S, Condori-Fernandez N, González A, Pastor Ó. Evaluating the completeness and granularity of functional requirements specifications: A controlled experiment. In 2009 17th IEEE International Requirements Engineering Conference 2009 Aug 30 (pp. 161-170). IEEE.
63. Ormandjieva O, Hussain I, Kosseim L. Toward a text classification system for the quality assessment of software requirements written in natural language. In 2007 international Workshop on Software Quality Assurance: in conjunction with the 6th ESEC/FSE joint meeting (pp. 39-45).
64. Shah US, Jinwala DC. Resolving ambiguity in natural language specification to generate UML diagrams for requirements specification. International Journal of Software Engineering, Technology and Applications. 2015;1(2-4):308-34.
65. Ramos R, Piveta EK, Castro J, Araújo J, Moreira A, Guerreiro P, Pimenta MS, Price RT. Improving the quality of requirements with refactoring. In 2007 Anais do VI Simpósio Brasileiro de Qualidade de Software (pp. 141-155). SBC.
66. Yang H, De Roeck A, Gervasi V, Willis A, Nuseibeh B. Speculative requirements: Automatic detection of uncertainty in natural language requirements. In 2012 20th IEEE International Requirements Engineering Conference (RE) 2012 Sep 24 (pp. 11-20). IEEE.
67. Arora C, Sabetzadeh M, Briand L, Zimmer F. Automated checking of conformance to requirements templates using natural language processing. IEEE Transactions on Software Engineering. 2015 May 1;41(10):944-68.
68. Wong B. A study of the metrics for measuring the quality of the requirements specification document. In 2004 International Conference on Software Engineering Research and Practice. CSREA Press.
69. Saito S, Takeuchi M, Yamada S, Aoyama M. RISDM: A requirements inspection systems design methodology: Perspective-based design of the pragmatic quality model and question set to SRS. In 2014 IEEE 22nd International Requirements Engineering Conference (RE) 2014 Aug 25 (pp. 223-232). IEEE.
70. Li FL, Horkoff J, Liu L, Borgida A, Guizzardi G, Mylopoulos J. Engineering requirements with desiree: An empirical evaluation. In 2016 Conference on Advanced Information Systems Engineering (pp. 221-238). Springer, Cham.
71. Sateli B, Angius E, Witte R. The reqwiki approach for collaborative software requirements engineering with integrated text analysis support. In 2013 IEEE 37th Annual Computer Software and Applications Conference 2013 Jul 22 (pp. 405-414). IEEE.
72. Heitmeyer CL, Jeffords RD, Labaw BG. Automated consistency checking of requirements specifications. ACM Transactions on Software Engineering and Methodology. 1996 Jul 1;5(3):231-61.
73. Yang H, De Roeck A, Gervasi V, Willis A, Nuseibeh B. Extending nocuous ambiguity analysis for anaphora in natural language requirements. In 2010 IEEE International Requirements Engineering Conference (pp. 25-34). IEEE.
74. Kiyavitskaya N, Zeni N, Mich L, Berry DM. Requirements for tools for ambiguity identification and measurement in natural language requirements specifications. Requirements Engineering. 2008 Sep 1;13(3):207-39.
75. Antinyan V, Staron M, Sandberg A, Hansson J. A complexity measure for textual requirements. In 2016 Joint Conference of the International Workshop on Software Measurement and the International Conference on Software Process and Product Measurement (IWSM-MENSURA) 2016 Oct 5 (pp. 148-158). IEEE.
76. El-Attar M, Miller J. Improving the quality of use case models using antipatterns. Software & Systems Modeling. 2010 Apr 1;9(2):141-60.
77. Liskin O, Pham R, Kiesling S, Schneider K. Why we need a granularity concept for user stories. In 2014 International Conference on Agile Software Development (pp. 110-125). Springer.
78. Stålhane T, Wien T. The DODT tool applied to sub-sea software. In 2014 IEEE 22nd International Requirements Engineering Conference (RE) 2014 Aug 25 (pp. 420-427). IEEE.
79. Verma K, Kass A, Vasquez RG. Using syntactic and semantic analyses to improve the quality of requirements documentation. Semantic Web. 2014 Jan 1;5(5):405-19.
80. Saito S, Takeuchi M, Hiraoka M, Kitani T, Aoyama M. Requirements clinic: Third party inspection methodology and practice for improving the quality of software requirements specifications. In 2013 21st IEEE International Requirements Engineering Conference (RE) 2013 Jul 15 (pp. 290-295). IEEE.
81. Chantree F, Nuseibeh B, De Roeck A, Willis A. Identifying nocuous ambiguities in natural language requirements. In 14th IEEE International Requirements Engineering Conference (RE’06) 2006 Sep 11 (pp. 59-68). IEEE.
82. Degiovanni R, Molina F, Regis G, Aguirre N. A genetic algorithm for goal-conflict identification. In Proceedings of the 33rd ACM/IEEE International Conference on Automated Software Engineering 2018 Sep 3 (pp. 520-531).
83. Nakatani T, Goto H, Nakamura T, Shigo O. A method to generate traverse paths for eliciting missing requirements. In Proceedings of the Australasian Computer Science Week Multiconference 2019 Jan 29 (pp. 1-10).
84. Ahmad S, Anuar U, Emran NA. A tool-based boilerplate technique to improve SRS quality: an evaluation. Journal of Telecommunication, Electronic and Computer Engineering (JTEC). 2018 Jul 5;10(2-7):111-4.
85. Osman MH, Zaharin MF. Ambiguous software requirement specification detection: An automated approach. In 2018 IEEE/ACM 5th International Workshop on Requirements Engineering and Testing (RET) 2018 Jun 2 (pp. 33-40). IEEE.
86. Anuar U, Ahmad S, Emran NA. An Empirical Investigation on a Tool-Based Boilerplate Technique to Improve Software Requirement Specification Quality. International Journal of Advanced Computer Science and Applications IJACSA. 2018 Dec 1;9(12):397-401.
87. Usdadiya C, Tiwari S, Banerjee A. An Empirical Study on Assessing the Quality of Use Case Metrics. In 2019 Innovations on Software Engineering Conference (pp. 1-11).
88. Kopczyńska S, Nawrocki J, Ochodek M. An empirical study on catalog of non-functional requirement templates: Usefulness and maintenance issues. Information and Software Technology. 2018 Nov 1;103:75-91.
89. Arora C, Sabetzadeh M, Briand LC. An empirical study on the potential usefulness of domain models for completeness checking of requirements. Empirical Software Engineering. 2019 Aug;24(4):2509-39.
90. Ferrari A, Esuli A. An NLP approach for cross-domain ambiguity detection in requirements engineering. Automated Software Engineering. 2019 Sep;26(3):559-98.
91. Ambroziewicz A, Śmiałek M. Applying use case logic patterns in practice: lessons learnt. In KKIO Software Engineering Conference 2018 Sep 27 (pp. 34-49). Springer, Cham.
92. Ko D, Kim S, Park S. Automatic recommendation to omitted steps in use case specification. Requirements Engineering. 2019 Dec;24(4):431-58.
93. Mokammel F, Coatanéa E, Coatanéa J, Nenchev V, Blanco E, Pietola M. Automatic requirements extraction, analysis, and graph representation using an approach derived from computational linguistics. Systems Engineering. 2018 Nov;21(6):555-75.
94. Halim F, Siahaan D. Detecting Non-Atomic Requirements in Software Requirements Specifications Using Classification Methods. In 2019 1st International Conference on Cybernetics and Intelligent System (ICORIS) 2019 Aug 22 (Vol. 1, pp. 269-273). IEEE.
95. Hasso H, Dembach M, Geppert H, Toews D. Detection of Defective Requirements using Rule-based Scripts. In 2nd Workshop on Natural Language Processing for Requirements Engineering (NLP4RE): in conjunction with REFSQ 2019.
96. Winter K, Femmer H, Vogelsang A. How Do Quantifiers Affect the Quality of Requirements?. In International Working Conference on Requirements Engineering: Foundation for Software Quality 2020. Springer, Cham.
97. Urbieta M, Torres N, Rivero JM, Rossi G, Dominguez-Mayo FJ. Improving mockup-based requirement specification with end-user annotations. In International Conference on Agile Software Development 2018 May 21 (pp. 19-34). Springer, Cham.
98. García-López D, Segura-Morales M, Loza-Aguirre E. Improving the quality and quantity of functional and non-functional requirements obtained during requirements elicitation stage for the development of e-commerce mobile applications: an alternative reference process model. IET Software. 2020 Apr;14(2):148-58.
99. Mezghani M, Kang J, Sèdes F. Industrial requirements classification for redundancy and inconsistency detection in SEMIOS. In 2018 IEEE International Requirements Engineering Conference (pp. 297-303). IEEE.
100. Wang Y, Wang T, Sun J. PASER: a pattern-based approach to service requirements analysis. International Journal of Software Engineering and Knowledge Engineering. 2019 Apr;29(04):547-76.
101. Dalpiaz F, Van der Schalk I, Lucassen G. Pinpointing ambiguity and incompleteness in requirements engineering via information visualization and NLP. In International Working Conference on Requirements Engineering: Foundation for Software Quality 2018 Mar 19 (pp. 119-135). Springer, Cham.
102. Ghannem A, Hamdi MS, Kessentini M, Ammar HH. Search-based requirements traceability recovery: A multi-objective approach. In 2017 IEEE Congress on Evolutionary Computation (CEC) 2017 Jun 5 (pp. 1183-1190). IEEE.
103. Tiwari S, Gupta A. Use case specifications: How complete are they?. Journal of Software: Evolution and Process. 2020 Jan;32(1):e2218.
104. Ahrens M, Schneider K. Using Eye Tracking Data to Improve Requirements Specification Use. In 2020 International Working Conference on Requirements Engineering: Foundation for Software Quality (pp. 36-51). Springer, Cham.
105. Hu W, Carver JC, Anu V, Walia GS, Bradshaw GL. Using human error information for error prevention. Empirical Software Engineering. 2018 Dec;23(6):3768-800.

## Footnotes

1 https://doi.org/10.5281/zenodo.5510222.

2 https://dl.acm.org/search/advanced.

3 https://ieeexplore.ieee.org/search/advanced.

4 https://www.sciencedirect.com/search.

5 https://link.springer.com/advanced-search.

6 https://apps.webofknowledge.com/.

7 https://scholar.google.de/.

8 Discussion of requirements is popular, but discussion of requirements engineering is not

9 https://www.mendeley.com/.

10 There are no issues of authority between R1 and R2.

11 “Experiment” and “controlled experiment” are two separate categories as we extracted the methodology “as stated”.

12 Note that both figures may look like they show similar magnitudes of values, but the log scale draws these values closer than they are.

13 In the replication package the granularity available from our extraction is finer.

14 The “elicitation scenario” was a formalised requirements elicitation process condensed into a formal model. As described by the authors, “the [model] consists of a combination of the four requirements elicitation techniques indicated above, combined in ten clearly defined steps” [S98].

15 “Correctness” is not listed in 29148-2011, but was again added in 29148-2018.

16 Our study scope and search string required explicit use of “quality” to find research targeted at improving quality, because in a broad sense, all RE research is trying to improve the state of RE. Our interest and the scope of this SMS, however, was on research that explicitly targeted quality.

17 https://doi.org/10.5281/zenodo.5510222.
